# Integrative Transcriptomic and Network-Based Analysis of Neuromuscular Diseases

**DOI:** 10.3390/ijms26199376

**Published:** 2025-09-25

**Authors:** Federico García-Criado, Lucia Hurtado-García, Elena Rojano, Álvaro Esteban-Martos, Jesús Pérez-García, Pedro Seoane, Juan A. G. Ranea

**Affiliations:** 1 Department of Molecular Biology and Biochemistry, Faculty of Sciences, University of Malaga, Bulevar Louis Pasteur, 31, 29010 Malaga, Spain; federogc98@uma.es (F.G.-C.); lhurtado@uma.es (L.H.-G.); alvaroesteban@uma.es (Á.E.-M.); jesuspg94@uma.es (J.P.-G.); seoanezonjic@uma.es (P.S.); ranea@uma.es (J.A.G.R.); 2 Instituto de Investigación Biomédica de Málaga y Plataforma en Nanomedicina (IBIMA-Plataforma BIONAND), C/ Severo Ochoa, 35, Parque Tecnológico de Andalucía (PTA), Campanillas, 29590 Málaga, Spain; 3 CIBER de Enfermedades Raras (CIBERER), Avda. Monforte de Lemos, 3-5, Pabellón 11, Planta 0, 28029 Madrid, Spain; 4 Instituto Nacional de Bioinformática (INB/ELIXIR-ES), Instituto de Salud Carlos III (ISCIII), C/ Sinesio Delgado, 4, 28029 Madrid, Spain

**Keywords:** neuromuscular diseases, integrative analysis, differential gene expression, network-based analysis

## Abstract

Neuromuscular diseases (NMDs) like Duchenne muscular dystrophy (DMD), limb–girdle muscular dystrophy (LGMD), and amyotrophic lateral sclerosis (ALS) are rare, progressive disorders with complex molecular mechanisms. Traditional transcriptomic analyses often struggle to capture systems-level dysregulation, especially given the small sample sizes typical of rare disease studies. Our differential expression analysis of eight public RNA-seq datasets from various cell types in DMD, LGMD, and ALS revealed not only disease-relevant pathways but also unexpected enrichments, such as renal development, suggesting systemic impacts beyond muscle tissue. To address limitations in capturing broader molecular mechanisms, we applied an integrative systems biology approach combining differential expression data, protein–protein interaction (PPI) networks, and network embedding techniques. Comparative functional enrichment revealed shared pathways, including glycosaminoglycan binding in both DMD and *FUS*-related ALS, implicating extracellular matrix–protein interactions in *FUS* mutation effects. Mapping DEGs onto the human PPI network and assessing their proximity to causal genes uncovered dysregulated non-coding RNAs, such as *PAX8-AS1*, *SBF2-AS1*, and *NEAT1*, potentially indicating common regulatory roles. We also found candidate genes within disease-proximal clusters, like *HS3ST3A1*, which may contribute to pathogenesis. Overall, this integrative approach reveals shared transcriptional programs and novel targets, advancing our understanding and potential treatment strategies for NMDs.

## 1. Introduction

Neuromuscular diseases (NMDs) encompass a diverse group of disorders that affect the function of muscles and the nerves that control them, often leading to progressive weakness, loss of motor function, and significant disability [1,2,3,4]. Among these, Duchenne muscular dystrophy (DMD) and amyotrophic lateral sclerosis (ALS) are two of the most extensively studied NMDs [5,6]. Although less prevalent than DMD or ALS, limb–girdle muscular dystrophy (LGMD) represents an important subgroup within the muscular dystrophies due to its phenotypic similarities with DMD, particularly in terms of progressive weakness affecting the shoulder and pelvic girdle muscles. While DMD is primarily characterized by progressive muscle degeneration due to mutations in the *DMD* gene [7], ALS involves the degeneration of motor neurons, with a multifactorial etiology involving both genetic and environmental factors [8]. Regarding LGMD, it encompasses a genetically heterogeneous set of disorders caused by mutations in various genes (including *CAPN3*, *TNPO3* and *LMNA*, among others) involved in muscle fiber integrity, membrane repair, or sarcomere organization [9,10]. Even the age of onset and rate of progression may vary; LGMD often mirrors the muscle degeneration patterns seen in DMD, which can be useful in studies exploring shared molecular mechanisms among diseases [11]. Despite the clinical and pathological differences between DMD and LGMD and ALS, these diseases have overlapping clinical features, including progressive muscle weakness and dysphagia [12], suggesting they may share common molecular pathways that remain poorly understood, contributing to delays in diagnosis and limited therapeutic options [13].

One of the significant challenges in the study of NMDs is the heterogeneity of their pathological phenotypes and the associated genetic profiles [14]. For instance, while DMD is typically linked to a single gene mutation [7], its clinical progression can vary due to modifier genes and environmental influences [15]. In contrast, ALS exhibits remarkable genetic heterogeneity [16], with mutations in over 40 genes implicated in its familial and sporadic forms [17]. Similar to ALS, LGMD includes multiple subtypes of the disease depending on the gene affected [9]. This complexity complicates the establishment of universal diagnostic biomarkers and therapeutic targets [18], resulting in prolonged diagnostic timelines and suboptimal treatment strategies. Moreover, the lack of comprehensive insights into the molecular mechanisms underlying these diseases hinders the development of effective interventions [1].

Identifying common molecular mechanisms and genetic factors shared by NMDs could provide critical insights into their pathogenesis and open new avenues for diagnostic and therapeutic advancements [1]. Transcriptomic studies, which analyze gene expression changes at the RNA level, offer a powerful approach to unravel the molecular underpinnings of these diseases [19,20]. Differential gene expression analysis, in particular, has been instrumental in identifying genes and pathways dysregulated in NMDs [20,21]. However, interpreting these data in isolation often falls short of capturing the complex interactions among genes and their regulatory networks.

To address this limitation, network-based approaches have emerged as complementary tools to integrate transcriptomic data and elucidate the relationships between differentially expressed genes (DEGs) [22]. By mapping DEGs onto biological networks, such as protein–protein interaction (PPI) networks, researchers can identify clusters of DEGs that are close to the disease-causal gene and determine pathways that could play central roles in disease pathogenesis [23]. More recently, the application of network embedding techniques has further advanced this field by enabling the transformation of complex network topologies into low-dimensional vector spaces that preserve neighborhood and connectivity information. In biological terms, these techniques allow us to convert a large, intricate interactome, comprising thousands of proteins and their interactions, into mathematical representations (vectors) where proteins with similar interaction patterns are placed close together in the new space. Unlike traditional clustering methods that rely uniquely on topological features, like node degree (number of direct connections between nodes) or direct neighbors (nodes directly connected to a node of interest), embeddings can capture higher-order structures, such as indirect connections or community contexts, which are often biologically meaningful. For example, two genes that are not directly connected but share similar interaction environments may be projected nearby in the embedded space, reflecting potential co-regulation or shared functional roles. These embeddings facilitate more accurate clustering, gene prioritization, and pathway inference, thereby improving the detection of functionally relevant modules and enhancing our capacity to pinpoint potential biomarkers and therapeutic targets in a systems-level context [24,25,26]. With these considerations, this integrative framework not only enhances the understanding of molecular mechanisms but also facilitates the prioritization of potential biomarkers and therapeutic targets.

In this study, we aim to leverage differential expression and network-based analyses to identify shared genetic factors and molecular mechanisms in DMD, LGMD, and ALS. By integrating RNA-seq data with PPI networks, we seek to uncover critical genes and pathways implicated in these diseases. Our findings have the potential to enhance the understanding of NMD pathogenesis, improve diagnostic accuracy, and inform the development of targeted therapies that address common aspects of these debilitating conditions.

## 2. Results

The results of the read pre-processing indicated good overall quality across all samples. In nearly all samples, the percentage of reads uniquely mapped to the reference genome was very high, with the exception of one control and one patient from the ALS_iN_C9ORF72 dataset (Supplementary Figure S1G). No signs of adapter contamination or major sequencing artifacts were found (Supplementary Figure S2). In addition, for each dataset, Supplementary Figure S3 illustrates the total number of reads remaining after pre-processing, the number of reads that aligned uniquely to the reference genome, and, among these, the subset that mapped to annotated genes.

### 2.1. Quality Assessment and Differential Expression Analysis Across Datasets

**Figure 1:**
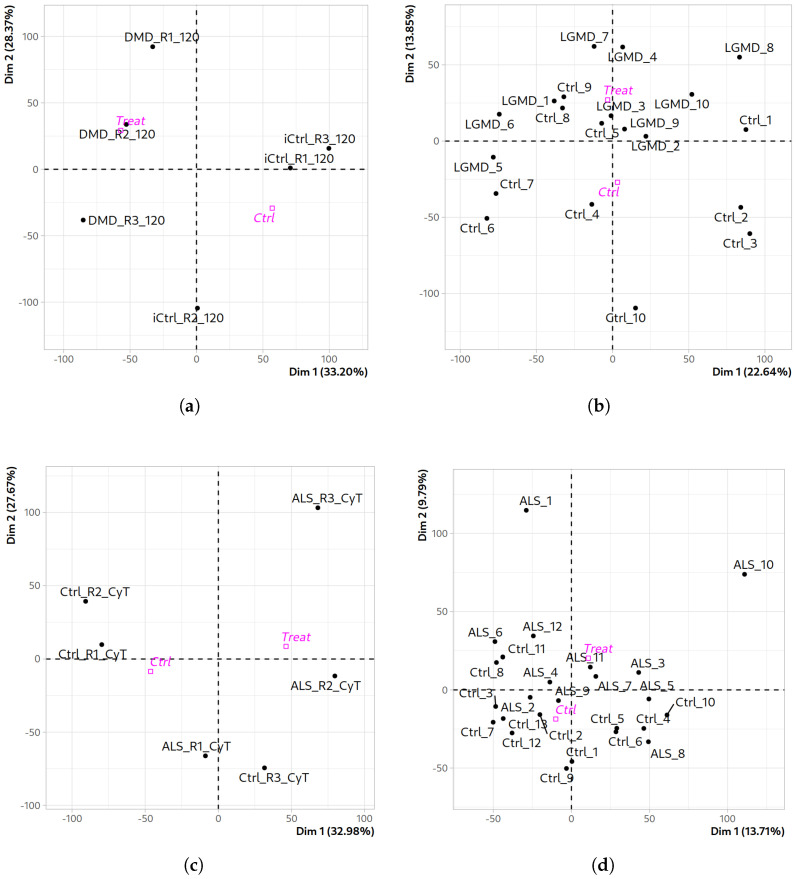
Principal component analysis (PCA) performed for the following datasets (each one representing a different causal gene): (**a**) DMD_myot (*DMD*), (**b**) LGMD_pbmc (*TNPO3*), (**c**) ALS_iN_C9ORF72 (*C9ORF72*), and (**d**) ALS_fib_FUS (*FUS*). The centroid of each group of samples compared (Ctrl and Treat) is displayed. DMD: Duchenne muscular dystrophy; LGMD: limb–girdle muscular dystrophy; ALS: amyotrophic lateral sclerosis; iCtrl: isogenic control. The centroid of the control and treatment groups is represented in magenta.

Differential expression analysis was performed with ExpHunter Suite independently for each neuromuscular disease (NMD) dataset. It includes a data quality control module that performs principal component analysis (PCA) to assess the distribution of samples and a Hierarchical Clustering on Principal Components (HCPC) that groups samples in clusters sharing similar characteristics. For the DMD datasets, in the case of the DMD_myot dataset (Figure 1a), it is noteworthy that the control iCtrl_R2_120 does not appear grouped with the rest of the controls in the principal component of the PCA, which accounts for 33.20% of the explained variability. In the HCPC of this comparison, we can see three well-differentiated clusters of samples: two with controls and patients perfectly grouped in their categories and another one including iCtrl_R2_120 along with the DMD_R3_120 patient sample (Supplementary Figure S5A). However, in the case of DMD_pCard (Supplementary Figure S4A), a clear separation of the samples between the two groups is observed: control (“ctrl”) and patient (“treat”). Specifically, the sample iCtrl_R1_RF is the one that is furthest from the control group, as can be seen in the HCPC (Supplementary Figure S5B). Regarding the DMD_cfib dataset, the PCA revealed certain separation between control and DMD patient samples (Supplementary Figure S4B); however, it is observed that Ctrl_1 and Ctrl_2 samples are somehow closer to DMD samples than to the rest of controls. This is also clearly observed in the HCPC (Supplementary Figure S5C), in which both controls are grouped with DMD samples in two different clusters, and Ctrl_3 and Ctrl_4 are included in another cluster. And in the case of the DMD_myob dataset, a clear separation between control and DMD samples is observed (Supplementary Figure S4C). Additionally, three distinguishable groups of patient samples are also observed. This is confirmed by the HCPC (Supplementary Figure S5D). These results are consistent with the original study, since three replicates were extracted from each individual.

Regarding the results obtained for the analysis of the datasets corresponding to LGMD, in the case of the LGMD_myob dataset, a clear separation is observed between the two groups of samples in the PCA (Supplementary Figure S4D), in which the principal component accounts for 96.38% of the explained variability, as well as a very close clustering between the samples that are part of the sick group, while there is more distance between the samples of the control group, with Ctrl_1 being the most distant. In the HCPC, this separation of samples is reflected in three distinct clusters, one of them including the patient samples and two with control samples (Supplementary Figure S6A). The LGMD_pbmc dataset (Figure 1b) exhibits substantial heterogeneity among its samples. The first principal component accounts for 22.64% of the explained variability and reveals the presence of distinct sample groupings, such as the control samples Ctrl_1, Ctrl_2, and Ctrl_3, and the patient samples LGMD_2, LGMD_3, and LGMD_10. This pattern is further supported by the dendrogram generated through HCPC analysis (Supplementary Figure S6B), which identifies up to seven distinct clusters: two clusters composed exclusively of control samples, three containing only patient samples, and two including a mix of both sample types.

Regarding the PCA results for ALS datasets, in the case of ALS_iN_C9ORF72 we carried out the comparison of controls vs. ALS patients for cytoplasmic samples. As can be seen in Figure 1c, there is a clear separation between control and patient groups, with the exception of the samples labeled R3. These do not cluster with their expected groups: the control R3 sample clusters with ALS patients, while the ALS R3 sample appears separated from both groups (Supplementary Figure S6C). In the case of the ALS_fib_FUS dataset (Figure 1d), PCA reveals a separation between both groups of samples (controls and FUS-mutated ALS samples) not very well defined between the control and patient groups on the basis of the second component, which accounts for 9.79% of the explained variability. Samples ALS_1 and ALS_10 are the most distant in the PCA space compared to the others; however, we chose not to exclude them from the analysis to maintain sample balance in the comparison. As observed in the HCPC for this dataset (Supplementary Figure S6D), the samples cluster into different groups. Samples ALS_1, ALS_3, ALS_6, and ALS_10 form clusters on their own. There are two clusters that include only control samples and one with four ALS patient samples and a final cluster that combines both sample types.

For each dataset, differential expression analysis was performed by comparing samples as specified in Section 4. Table 1 summarizes the number of differentially expressed genes (DEGs) identified in each dataset, specifying how many were over-expressed and how many were down-regulated based on the log_2_ fold change (log_2_FC). Supplementary Tables S1 and S2 present, for DMD and for LGMD and ALS, respectively, the top five over-expressed and down-regulated genes identified in each dataset, ranked by their log_2_FC values.

Overall, the average number of expressed genes across the eight datasets is 13,429.1. Among these, a higher number of expressed genes is observed in samples taken from different tissues, like iNeurons, cardiac fibroblasts, and myoblasts, than from PBMC and fibroblasts (Table 1). Likewise, a higher number of over-expressed genes is observed than down-regulated genes, except in the case of DMD_pcard and LGMD_myob, where 91.3% and 63.6% of DEGs, respectively, have negative log_2_FC values.

Differential expression analysis across the DMD and LGMD datasets revealed both dataset-specific and, in some cases, shared transcriptional alterations. Notably, a subset of the most significantly expressed genes is common to different datasets, suggesting potentially conserved molecular signatures. For instance, *MEG3* and ENSG00000225746 are over-expressed in both DMD_myot and DMD_myob; *PAX8-AS1* appears among the top over-expressed genes in DMD_pCard and DMD_cfib; *GSTM1* and *TRPV2* are shared between DMD_myob and LGMD_myob; and *SFRP4* is down-regulated in both DMD_cfib and ALS_fib_FUS. The results of the functional enrichment analysis for each dataset, presented as over-representation analysis (ORA) graphs generated by ExpHunter Suite, are shown in Supplementary Figure S7. In DMD_myot and DMD_myob, DEGs were enriched in biological processes related to renal system development, synaptic organization, and immune responses, including type I interferon signaling and hypoxia response. In contrast, DMD_cfib (the dataset with the fewest DEGs) had more limited enrichment, mainly involving extracellular matrix organization and nutrient response. In DMD_pCard, over-expressed genes, such as *PAX8-AS1* and *GJD2*, were linked to functions in connective tissue development, ossification, coagulation, and nervous system regulation. In LGMD_myob, strong over-expression of *ACSM5* and *GSTM1* reflected robust enrichment in muscle-specific processes, including muscle cell differentiation, sarcomere organization, and cardiac function. On the other hand, LGMD_pbmc showed a distinct immune-related signature, with DEGs involved in chemokine response, leukocyte migration, and immune cell activation, consistent with high expression of *MMP1*, *CXCL5*, and *SERPINB2*. The two ALS datasets also showed contrasting profiles. ALS_iN_C9ORF72 exhibited over-expression of *TUNAR* and *TMEM132E*, with enriched functions overlapping with those observed in DMD_myot, including renal morphogenesis, axon guidance, and glial differentiation. In contrast, ALS_fib_FUS displayed more moderate expression changes and lacked enrichment for neuronal processes. Instead, functional categories were related to cardiac muscle tissue growth, blood circulation, and mesenchyme morphogenesis, suggesting a fibroblast-specific response.

#### Comparative Functional Profiling Across Neuromuscular Disease Transcriptomes

**Figure 2:**
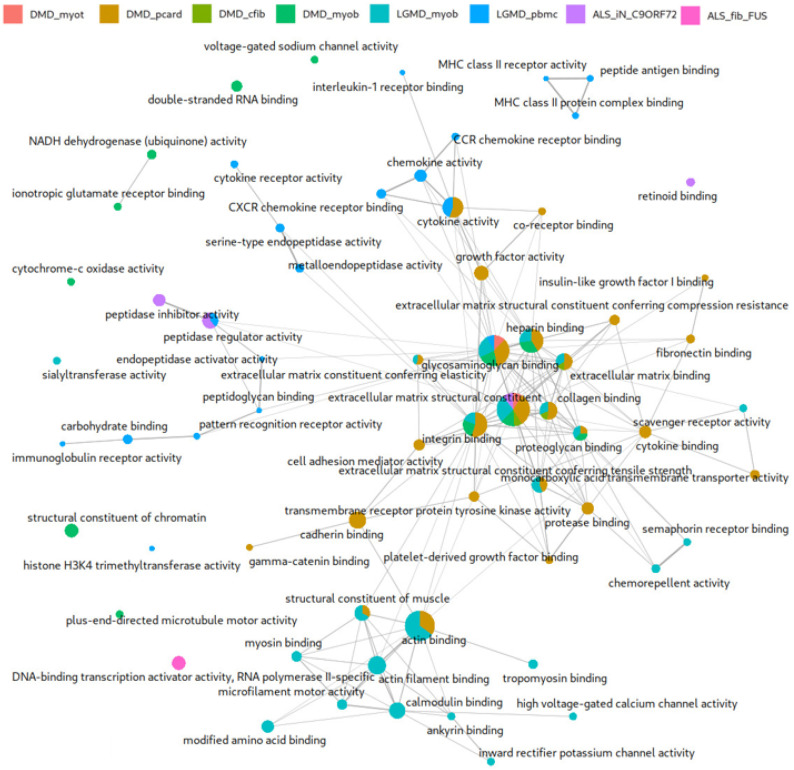
Comparative functional enrichment network. It represents the top enriched functional categories, specifically Gene Ontology (GO) biological process terms, identified across the different datasets (DMD_myot, DMD_pCard, DMD_cfib, DMD_myob, LGMD_myob, LGMD_pbmc, ALS_iN_C9ORF72, and ALS_fib_FUS). Nodes correspond to GO biological process terms (when both parent and child terms are present in the network, only the child terms are retained, and the parent terms are removed), and edges indicate functional relationships based on shared DEGs. Edge thickness reflects the number of shared DEGs connecting two GO terms, with thicker edges denoting stronger functional overlap.

To explore the functional relationships among DEGs across NMDs, we used ExpHunter Suite to perform a comparative functional enrichment analysis (Figure 2). This approach enabled us to identify enriched Gene Ontology (GO) biological processes associated with each dataset to determine which functional categories are shared among different NMDs and which are unique to specific conditions. We used a *p*-value of 0.05 to select significant annotations.

There are several functional categories shared among the eight datasets, as well as clusters specific to each of them, which share some connection with more general functions, establishing a highly interconnected network. Exceptionally, some isolated functions are observed, generally belonging to the same dataset. The functional categories extracellular matrix binding and glycosaminoglycan binding are shared by most of the datasets. Other categories, such as actin binding and structural constituent of muscle, are shared by both LGMD_myob and DMD_pCard, and both functions connect directly with other, more dataset-specific functions, such as filament actin binding and calmodulin binding, specific to the LGMD_myob dataset, and with cadherin binding and cytokine binding, specific to the DMD_pCard dataset. There are also shared functions among several datasets of different diseases taken from similar tissues, such as integrin binding for DMD_myob, LGMD_myob, and DMD_pCard and growth factor binding and collagen binding for the DMD_pCard, DMD_cfib, and LGMD_myob datasets. It is also worth noting that some of the functions associated with the DEGs of DMD_myob, LGMD_myob, and LGMD_pbmc form clusters of functions characterized exclusively for those datasets. Also noteworthy is the peptidase regulator activity function, shared between the LGMD_pbmc and ALS_iN_C9ORF72 datasets, belonging, respectively, to two very different NMDs in phenotypic terms.

### 2.2. Mapping Differential Expression Profiles onto the Human Interactome

**Figure 3:**
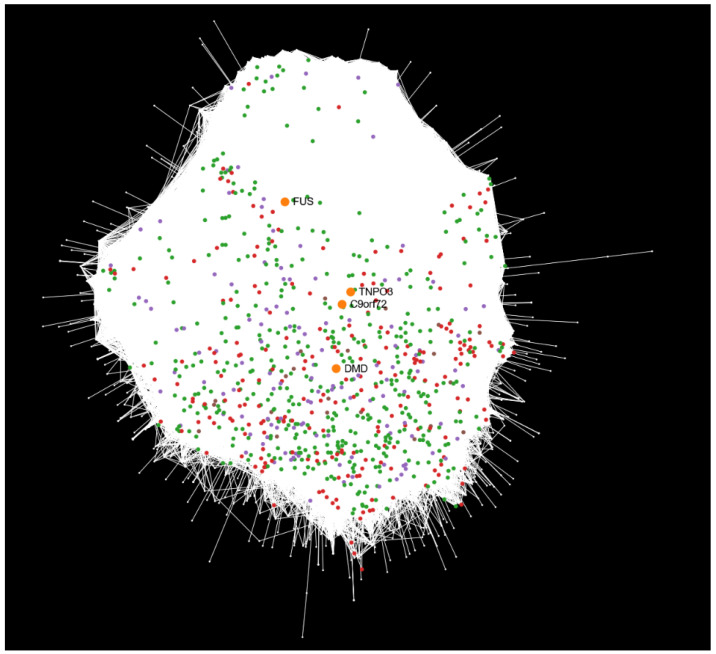
Protein–protein interaction network belonging to the dataset view, based on the union of all expressed genes across all the datasets (white body). Each differentially expressed gene list is represented in different colors, depending on the dataset to which they belonged. Those from the four DMD-related and the two LGMD-related datasets were collapsed into two aggregated DEG lists for visualization clarity. Green: DMD-related datasets; red: LGMD-related datasets; purple: ALS_iN_C9ORF72 dataset; and brown: ALS_fib_FUS dataset. Disease-causal genes (*DMD*, *TNPO3*, *C9ORF72*, and *FUS*) for all datasets are represented as orange nodes.

Combining protein–protein interactions from all datasets allows the construction of a comprehensive network capturing interactions consistently present across multiple NMD models, providing a global perspective on how disease-causal genes relate to the observed differential expression profiles and enabling the identification of shared modules, conserved pathways, and core disease mechanisms that might not be apparent in individual datasets. We constructed a unified network composed of all expressed genes across the eight datasets (Figure 3). The network layout is generated using an attraction–repulsion algorithm that organizes nodes by connectivity: highly connected hubs cluster toward the center, while barely connected or isolated nodes are displaced to the periphery. We will refer to this network as the dataset view to differentiate it from other representations used in this study. In this dataset view representation, DEGs from each dataset were mapped onto the human protein–protein interaction network. To improve visualization clarity, DEGs from the four DMD-related datasets (linked to the *DMD* gene) and the two LGMD-related datasets (linked to *TNPO3*) were merged into single DEG sets. Additionally, the four disease-causal genes corresponding to each NMD were used as seed nodes (starting reference points in the network) to explore and analyze the relationships between these causal genes and the DEGs. For further details and a complete network exploration, the interactive report is available in the integrative analysis results repository https://github.com/ElenaRojano/INTRINSED_datasets/blob/main/network_all_SAFE.html (accessed on 27 July 2025).

This integrated dataset view representation reveals notable patterns of proximity among disease-causal genes. Interestingly, despite being linked to distinct disorders, *C9ORF72* (ALS) and *TNPO3* (LGMD) are positioned closely within the network. The DEGs surrounding these two genes primarily originate from the DMD- and LGMD-related datasets (Figure 3, green and red nodes, respectively), rather than from ALS_iN_C9ORF72 (Figure 3, purple nodes). In the case of *TNPO3*, this is consistent with the phenotypic similarities between DMD and LGMD, both phenotypically similar NMDs [11]. Conversely, *DMD* is situated closer to *C9ORF72* than to *TNPO3*, despite the stronger phenotypic resemblance between DMD and LGMD. In addition, *FUS* (also associated with ALS) appears distant from *C9ORF72* in the network, suggesting that they may be involved in distinct molecular pathways or operate within separate topological contexts. Interestingly, the disease-causal gene *DMD* shows a marked spatial separation from its nearest DEGs in the interaction network, in contrast to the other three disease-causal genes, which are surrounded by DEGs in close proximity. In addition, the distribution of DEGs in the ALS_fib_FUS dataset is particularly striking, as the disease-causal gene *FUS* is spatially distant from its associated DEGs (Figure 3, brown nodes), which instead appear closer in the network to the *DMD* gene.

To consolidate this information, Table 2 summarizes the integrative analysis across all datasets, including the number of DEGs successfully mapped to the human protein–protein interaction network. Notably, the number of mapped DEGs varies substantially between datasets. For example, DMD_pCard (247 mapped DEGs) and DMD_myob (239) showed the highest number of DEGs mapped onto the interactome. LGMD_myob and ALS_iN_C9ORF72 also showed substantial mapping, with 206 and 127 DEGs, respectively. In contrast, datasets such as DMD_myot (34), DMD_cfib (13), LGMD_pbmc (19), and ALS_fib_FUS (21) exhibited much lower numbers of mapped DEGs. It is worth noting that these datasets yielded fewer DEGs in the differential expression analysis compared to the other datasets (all with ≥300 DEGs, Table 1), which partly explains their lower number of mapped genes.

#### Analysis of Unmapped Differentially Expressed Genes

Looking closely at the information provided in Table 1 on the total DEGs for each dataset, there are a number that do not map against the human interactome network. In this work, we referred these DEGs as *unmapped differentially expressed genes* (uDEGs). The reasons why these DEGs are left out of the network may be either due to the absence of known interactions or annotation limitations. uDEGs are classified into three categories: coding uDEGs, which are protein-coding genes not represented in the interaction network; non-coding RNAs (ncRNAs), which mainly include long non-coding RNAs (lncRNAs); and pseudogenes.

In our study, ncRNAs were the most represented category among uDEGs in all datasets, followed by pseudogenes and unmapped coding genes. The datasets DMD_myob and LGMD_myob presented the highest numbers of uDEGs, with 164 and 120, respectively. In both cases, the majority of uDEGs were ncRNAs (114 and 85, respectively), followed by pseudogenes and unmapped coding genes. Similarly, ALS_iN_C9ORF72 (50 uDEGs) and ALS_fib_FUS (36 uDEGs) also showed a predominance of ncRNAs (40 and 25, respectively), suggesting a potential regulatory contribution of these transcripts in ALS pathology. In contrast, datasets such as DMD_myot (17 uDEGs), DMD_cfib (14 uDEGs), and DMD_pCard (31 uDEGs) had a lower number of unmapped genes overall, with ncRNAs still representing the majority. LGMD_pbmc, despite its relatively low number of mapped DEGs (19), had a notable 53 uDEGs, including 19 ncRNAs and 19 pseudogenes, suggesting a potential enrichment of non-coding elements in this dataset. In Supplementary Table S4 we include all the uDEGs shared between datasets. Most of them are lncRNAs. For example, *PAX8-AS1* exhibits a striking expression pattern: down-regulated in DMD myoblasts (log_2_FC = −4.97) but consistently over-expressed in all other DMD datasets, as well as in the LGMD_myob dataset (log_2_FC = 4.69). A similar pattern is observed for *MEG3*, which is strongly over-expressed in DMD_myot (log_2_FC = 9.32) and DMD_myob (log_2_FC = 8.56) but down-regulated in DMD_pCard (log_2_FC = −2.16). Notably, *SBF2-AS1* and *NEAT1* emerged as differentially expressed lncRNAs in different diseases and tissues, but with opposing expression patterns: *SBF2-AS1* was over-expressed in LGMD_myob (log_2_FC = 3.71) yet down-regulated in ALS_iN_C9ORF72 (log_2_FC = −2.26), while *NEAT1* was over-expressed in DMD_myob (log_2_FC = 2.15) and down-regulated in ALS_fib_FUS (log_2_FC = −1.46). No differentially expressed ncRNAs were found in common between LGMD_pbmc and the rest of the datasets.

### 2.3. Mapping Disease-Associated Transcriptional Profiles onto the Human Interactome

**Figure 4:**
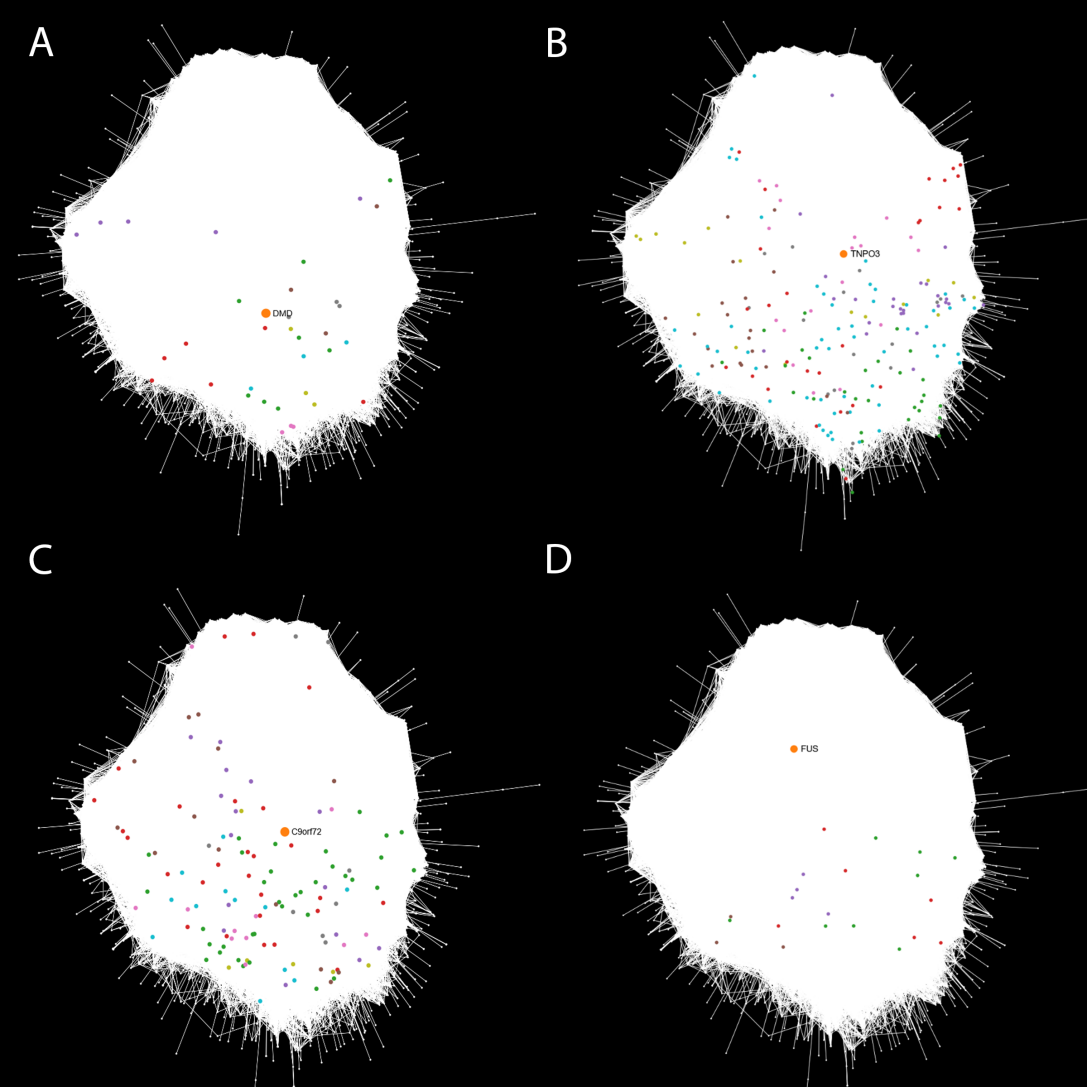
Protein–protein interaction networks from the disease view representation. Colored nodes correspond to clusters of differentially expressed genes (DEGs) for each dataset. Orange dots represent the disease-related gene used as a reference. (**A**) DMD_myot (*DMD*), (**B**) LGMD_myob (*TNPO3*), (**C**) ALS_iN_C9ORF72 (*C9ORF72*), and (**D**) ALS_fib_FUS (*FUS*).

To gain deeper insights into the molecular mechanisms underlying NMDs, we performed a network-based integrative analysis by projecting the DEGs from each dataset onto the human protein–protein interaction network. We refer to this representation as the disease view to distinguish it from the previously described dataset view. In this context, we define *functional groups* as clusters of DEGs that interact closely with one another in the network and are also connected to the disease-associated gene specific to each dataset: *DMD* for the DMD_myot, DMD_pCard, DMD_cfib, and DMD_myob datasets, *TNPO3* for both LGMD_myob and LGMD_pbmc datasets, *C9ORF72* for the ALS_iN_C9ORF72 dataset, and *FUS* for the ALS_fib_FUS dataset. For simplicity, we selected representative datasets for each disease: DMD_myot, LGMD_myob, ALS_iN_C9ORF72, and ALS_fib_FUS (Figure 4A–D, respectively). The rest of the datasets are included in Supplementary Figure S8. For each of the datasets, the report with the interaction network resulting from the integrative analysis and the functional analysis of the clusters that will be detailed in the following sections is available in the results repository at https://github.com/ElenaRojano/INTRINSED_datasets/blob/main/network_all_SAFE.html (accessed on 27 July 2025).

In the case of the DMD-related datasets, a pairwise pattern of similarity emerges. The DMD_myot dataset (Figure 4A) shows clusters with fewer DEGs that are more widely dispersed across the network. A similar distribution is observed in the DMD_cfib dataset (Supplementary Figure S8B), which also displays a reduced number of clusters. This is consistent with the low number of DEGs identified in this dataset (80; see Table 1), although some remain in close proximity to the disease-causal gene *DMD*. In both datasets, DEGs exhibit a dispersed pattern throughout the network, potentially suggesting a more heterogeneous or less coordinated transcriptional response. In the case of DMD_pCard and DMD_myob (Supplementary Figure S8A,C), both display comparable network organizations, with multiple DEG clusters positioned near the disease-causal gene *DMD*, suggesting a more coherent functional or topological response to disease perturbation in these tissues. A similar pattern to that observed in the DMD_myot and DMD_cfib datasets was found in LGMD_pbmc (Supplementary Figure S8D), which contains a relatively low number of DEGs (160) compared to LGMD_myob (Figure 4B). This difference likely contributes to the reduced number of clusters detected in LGMD_pbmc. In LGMD_myob, DEGs are more heterogeneously distributed across the network, with several clusters forming around the disease-causal gene *TNPO3*, as well as additional clusters located in more peripheral regions. In contrast, LGMD_pbmc displays two smaller DEG clusters located at greater topological distances from *TNPO3*, although some genes within another cluster appear relatively close to the disease-associated gene in the network. Regarding the ALS-related datasets, ALS_iN_C9ORF72 (Figure 4C) exhibits a higher number of clusters with a denser distribution of DEGs compared to ALS_fib_FUS (Figure 4D). In this ALS_fib_FUS dataset, DEGs are strikingly distant from the disease-causal gene *FUS* within the interactome, reinforcing the observation from the dataset view that these genes occupy separate topological regions in the network.

These intra-group analyses reveal notable differences in the network organization of DEGs across datasets associated with the same disease gene. When comparing across disease groups, particularly between DMD, LGMD, and ALS datasets, distinct spatial patterns emerge: DMD-related datasets tend to have more compact DEG neighborhoods around *DMD*, while ALS datasets, especially ALS_fib_FUS, show more scattered DEG distributions. Furthermore, analyzing the distribution of the number of clusters and the number of DEGs per cluster provides insight into the heterogeneity and connectivity patterns of disease-associated expression changes within the network. In Table 2, we observe the highest number of DEG clusters (17) for the DMD_pCard dataset, followed by LGMD_myob (15) and DMD_myob (13) datasets, which also exhibited a substantial number of clusters. The DMD_cfib, LGMD_pbmc, and ALS_fib_FUS datasets showed the lowest number of clusters (4). Certainly, it is observed that datasets derived from non-specific tissues, like fibroblasts (DMD_cfib and ALS_fib_FUS) and PBMCs (LGMD_pbmc), show a lower number of DEGs per cluster compared to those obtained from specific tissues. For example, as can be observed in Supplementary Figure S10B, the median number of genes per cluster is 3 for DMD_myot and DMD_cfib and 5 for ALS_fib_FUS, whereas in DMD_myob the median rises to 20 and in DMD_pCard to 15.

Regarding the distribution of DEGs per cluster, in the four DMD datasets (Supplementary Table S15) we observe a lower number of DEGs per cluster in the datasets with a lower number of clusters (DMD_myot and DMD_cfib), compared to the DMD_pCard and DMD_myob datasets. Interestingly, in these last two datasets, the first two clusters have the same number of DEGs (32 and 29, respectively, in clusters 0 and 1), but there are no shared genes between any of the clusters between the different datasets. In the case of the LGMD datasets (LGMD_myob and LGMD_pbmc, Supplementary Table S16), more clusters and a higher abundance of DEGs are observed in the LGMD_myob dataset. This dataset, along with DMD_myob, both from samples of the same cell type (myoblasts), are the ones that returned the most DEGs in the differential expression analysis. And, despite not being the cluster with the highest number of DEGs, the ALS_iN_C9ORF72 dataset has the cluster with the most DEGs per cluster (35).

### 2.4. Embedding-Based Prioritization of DEG Clusters Relative to the Disease-Causal Gene

Given the complexity of the network structure, considering the large number of DEG-derived clusters and the substantial set of isolated genes, traditional topological measures may be insufficient to capture the nuanced relationships required to identify disease-relevant genes [27]. Classical approaches often rely on direct connectivity and may overlook functionally important nodes that are not topologically central or well-connected but may still be biologically proximal to the disease-causal gene.

In this context, we adopt a network embedding strategy to project the high-dimensional interactome into a continuous latent space, which enables a more sensitive assessment of gene proximity and reflects functional similarity beyond direct edges. Importantly, embedding allows us to prioritize both clustered and non-clustered (or “isolated”) DEGs according to their spatial proximity to the disease-causal gene under the hypothesis that genes closely located in the embedded space are more likely to participate in shared biological processes or pathways relevant to disease mechanisms. Thus, embedding provides a robust framework for integrative prioritization in complex disease networks where gene interactions may be indirect or context-dependent.

#### 2.4.1. DEG Cluster Priorititazion and Functional Analysis

**Figure 5:**
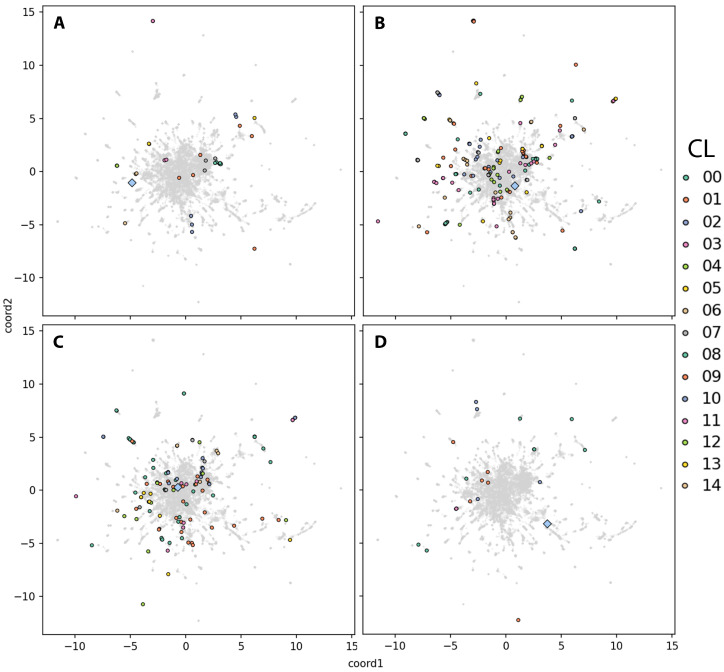
Two-dimensional embedding of gene interaction networks. Each panel represents the reduced vector space (coord1 vs. coord2) of the full gene interaction network based on STRING “Experimental” evidence. Background grey dots correspond to all expressed genes included in the network. The disease-causal gene (seed) for each condition (*DMD*, *TNPO3*, *C9ORF72*, and *FUS*) is represented as a blue rhombus. Panels correspond to the DMD_myot (**A**), LGMD_myob (**B**), ALS_iN_C9ORF72 (**C**), and ALS_fib_FUS (**D**) datasets. Colored dots represent differentially expressed genes (DEGs), grouped by cluster (CL).

Applying network embedding to each dataset reveals a higher number of clusters and mapped DEGs in datasets derived from differentiated tissues, such as LGMD_myob (Figure 5D), ALS_iN_C9ORF72 (Figure 5C), DMD_pCard (Supplementary Figure S9A), and DMD_myob (Supplementary Figure S9C). In contrast, a lower number of clusters is observed in the datasets that are of undifferentiated tissues: DMD_cfib (Supplementary Figure S9B), LGMD_pbmc (Supplementary Figure S9D), and ALS_fib_fus (Figure 5D). It is interesting to remark that, in the case of both DMD_myot and DMD_myob datasets (Supplementary Figure S9B and D, respectively), a noticeable spatial separation between the disease-causal gene (*DMD*) and the surrounding DEGs in the closest clusters is observed. This trend can be also seen in the LGMD_pbmc (Supplementary Figure S9D) and ALS_fib_FUS (Figure 5D) datasets. The opposite trend is observed for the rest of datasets (all from differentiated tissues). For example, in the case of both DMD_pCard and ALS_iN_C9ORF72 datasets (Supplementary Figure S9A and Figure 5C, respectively), DEGs in the clusters nearest to the respective causal genes (*DMD* and *C9ORF72*) are placed in a similar region within the interaction network, suggesting closer functional relationships. Furthermore, the number of genes per cluster is clearly higher in these tissue-specific datasets compared to the others.

To avoid possible biases in the visual representation of the data and the possibility of overlapping of the causal gene with any cluster, in Supplementary Tables S5 and S6 the ranked information, based on their similarity score, of the clusters closest and furthest to the causal gene is included. With this information, we can confirm that the closest cluster with respect to the *DMD* causal gene is observed for the DMD_cfib (cluster 0, score: 1.00) and DMD_pCard (cluster 8, score: 1.00) datasets, closely followed by DMD_myob (cluster 7, score: 0.94). In the case of DMD_myot, cluster 3 is the one with the highest clustering score for this dataset (score: 0.77). The rest of the clusters, except for cluster 10 for the DMD_myob dataset (score: 0.11), present score values very close to 0. No clusters are observed that could be relevant for the LGMD-related datasets and their association with the disease causal gene (*TNPO3*), and in ALS, cluster 9 of the ALS_iN_C9ORF72 dataset has a score of 0.31, a substantially lower clustering score value compared to those calculated for the mentioned DMD datasets. In the fibroblast-derived ALS dataset, cluster 2 is the closest to the *FUS* causal gene, with a similarity score of 0.02, the lowest among the four datasets for the cluster nearest to its corresponding causal gene. The close proximity of these clusters to the causal gene suggests that the associated DEGs may interact directly with it and be involved in related biological processes. Based on this assumption, we performed a functional enrichment analysis of the DEGs within each cluster, prioritizing those closest to the causal gene.

The functional enrichment analysis of DEGs in cluster 0 (*DMD* and *ANLN*) for the DMD_cfib dataset revealed a strong association with molecular functions central to cytoskeletal organization and muscle integrity (Supplementary Table S8). Among the significantly enriched terms were dystroglycan binding, nitric-oxide synthase binding, vinculin binding, actin binding, and structural constituent of muscle. These functions are closely related to the biological role of dystrophin. It is interesting to mention that in the comparison performed for the differential expression analysis of this dataset, the *DMD* gene appears strongly down-regulated (log_2_FC = −2.12). In the case of the DMD_pCard dataset (Supplementary Table S9), cluster 8 included genes enriched in biological processes related to the negative regulation of coagulation and hemostasis, including negative regulation of blood coagulation, negative regulation of hemostasis, and innervation among them. These functions may suggest that dystrophin deficiency in cardiac tissue may also influence vascular homeostasis and neural regulation. Functional analysis of cluster 7 DEGs in the DMD_myob dataset included key molecular functions associated with extracellular matrix organization and protein processing, like dystroglycan binding, apolipoprotein binding, extracellular matrix structural constituent, serine-type endopeptidase activity, and serine-type peptidase activity (Supplementary Table S10). These functions suggests a possible role for this cluster of DEGs in maintaining tissue structure and regulating proteolytic activity, processes that are often dysregulated in DMD. For the DMD_myot dataset, cluster 3 included DEGs enriched in terms related to cardiac and muscle function, including the regulation of cardiac muscle cell contraction, specifically in ventricles, bundle of His cell-to-Purkinje myocyte communication, and actin-mediated cell contraction (Supplementary Table S7). These processes are critical for maintaining coordinated cardiac conduction and contractile function, suggesting that genes in this cluster may reflect the broader impact of dystrophin deficiency beyond skeletal muscle, potentially contributing to the cardiomyopathic features frequently observed in DMD. Regarding the ALS datasets, in the case of ALS_iN_C9ORF72 we found that genes in cluster 9 were enriched in biological functions related to synaptic structure modification and mitochondrial apoptotic processes, like modification of postsynaptic structure, positive regulation of release of cytochrome c from mitochondria, and apoptotic mitochondrial changes (Supplementary Table S11). These results suggest a disruption in both neuronal connectivity and mitochondrial integrity in *C9ORF72*-associated ALS. And in the case of the ALS_fib_FUS dataset (Supplementary Table S12), cluster 2 DEGs were enriched in biological process that are mainly related to connective and adipose tissue development, Wnt signaling regulation, and pattern specification, which may point to potential alterations in mesenchymal tissue remodeling and developmental signaling pathways and contribute to disease mechanisms in ALS linked to *FUS* mutations.

#### 2.4.2. Prioritization of Isolated DEGs

In addition to clustered DEGs, we also identified a subset of network-mapped DEGs that do not form significant interactions with other DEGs and thus remain outside of defined functional clusters. We refer to these as “isolated DEGs”. Despite their lack of clustering, these genes may still play important roles in disease pathogenesis, particularly if they are located in close proximity to the disease-causal gene within the human interactome, as this spatial closeness can suggest potential functional relevance through shared or convergent biological processes.

The top ten isolated DEGs for each dataset are ranked by their similarity score, from highest to lowest, and included in Supplementary Table S13 for the DMD-related datasets and in Supplementary Table S14 for LGMD and ALS datasets. In the case of the DMD_myot dataset, the *DMD* gene, which is the causal gene for DMD, is excluded from the identified gene clusters. This is an intriguing observation, as it suggests that despite being central to the disease, *DMD* may not directly interact with the rest of DEGs. It is interesting that the gene *CDH12* appears shared between two datasets of the same disease, DMD_pCard and DMD_cfib, but even more so is the case of *HS3ST3A1*, which appears shared in the datasets LGMD_myob and ALS_fib_FUS, both belonging to two different diseases caused by mutations in different genes. In the case of the DMD_myob dataset, it should also be noted that the *EPSTI1* gene appears with a very high score (0.99), and, despite not having clustered with other DEGs, this fact suggests some kind of functional relationship. In addition, it is interesting to mention *ABCB11* and *PTPRN* genes in DMD_pCard and DMD_cfib, respectively, as the isolated DEGs with the highest scores for these two DMD datasets. In the LGMD_myob dataset, the high-scoring isolated genes *ST6GAL1* (score: 0.99), *SRPK3* (score: 0.96), and *P2RX5* (score: 0.89) may reflect downstream effects of *TNPO3* dysfunction. In the case of the LGMD_pbmc dataset, the isolated genes *CHERP* (score: 0.84) and *EPHA1* (0.82) may also represent functionally relevant downstream targets of *TNPO3*-related dysregulation due to its proximity in the network to the causal gene of LGMD. And regarding the DEGs that did not cluster in the ALS datasets, the proximity of *NREP* (neuronal regeneration-related protein, score: 0.93) to *C9ORF72* in the network may reflect shared roles in neuronal plasticity, cytoskeletal remodeling, and cellular stress responses. In addition, the second closest DEG to *C9ORF72* is *MAP1LC3C*, which encodes a protein involved in the regulation of microtubule dynamics and autophagy. Finally, the isolated genes *JMJD6* and *VRK1*, in the ALS_fib_FUS dataset, showed high similarity scores with *FUS* in the interaction network, suggesting potential functional connections.

## 3. Discussion

Understanding neuromuscular diseases (NMDs), such as Duchenne muscular dystrophy (DMD), limb–girdle muscular dystrophy (LGMD), and amyotrophic lateral sclerosis (ALS), requires a comprehensive exploration of their genetic and molecular bases. They are severe NMDs with distinct genetic origins: mutations in the *DMD* gene for DMD [7], different genes depending on the LGMD subtype (like *TNPO3* [28]), and alterations in more than 40 genes in ALS [16], including *C9ORF72* [29,30] and *FUS* [31,32] among the most common ALS-associated genes, along with *SOD1* and *TARDBP* [33]. Despite their phenotypic differences, they converge in the disruption of fundamental biological processes, including cellular homeostasis, inflammation, and synaptic function [11,34]. Thus, comprehending the molecular basis of these NMDs is crucial for the development of targeted and effective therapeutic strategies [1].

From a molecular perspective, transcriptomic analysis has proven to be a powerful approach for identifying gene expression alterations associated with neuroinflammatory and neurodevelopmental diseases [35], offering valuable insights into dysregulated pathways and potential biomarkers [36]. In the context of DMD, LGMD, and ALS, transcriptomic analysis through differential gene expression (RNA-seq) is crucial to elucidate the downstream molecular consequences of genetic alterations in key disease-associated genes, such as *DMD* [37,38], *TNPO3* [28], *C9ORF72* [29,39], and *FUS* [32], which may lead to altered expression profiles contributing to pathogenesis. First of all, to perform this study, we made sure to have at least two datasets for each of the diseases we analyzed, and we also performed sample selection from different tissue types to ensure biological relevance and enhance the interpretability of transcriptomic signatures. With this consideration, we selected samples from differentiated tissues, such as myoblasts (in the case of the DMD_myob and LGMD_myob datasets), cardiac fibroblasts (DMD_cfib dataset), iNeurons (ALS_iN_C9ORF72), and myotubes derived from reprogrammed skin fibroblasts (DMD_myot), and undifferentiated tissue samples, including fibroblasts, as in the DMD_cfib (cardiac fibroblasts) and ALS_fib_FUS datasets, and peripheral blood mononuclear cells (PBMCs) in the LGMD_pbmc dataset. Fibroblasts serve as a useful reference due to their accessibility and broad transcriptomic representation but may not fully capture tissue-specific pathological mechanisms. Therefore, we included cardiomyocytes for DMD, given the cardiac involvement commonly associated with dystrophin deficiency [40], and induced neurons (iNeurons) for ALS, as they more closely reflect the neurodegenerative processes central to the disease [41]. In addition, we incorporated datasets derived from myoblasts and myotubes to better characterize muscle-specific alterations in DMD and LGMD [42] and PBMCs to investigate systemic immune responses in LGMD [43], thus ensuring a broader representation of disease-relevant biological contexts. This strategy enabled a comprehensive view of both shared and tissue-specific alterations across NMDs, despite the challenge of limited high-quality data due to their rarity and our selection criteria.

### 3.1. Differential Expression Analysis Reveals Novel Insights from NMD Dataset-Specific Comparisons

Our differential expression results largely aligned with original studies, supporting the robustness of our pipeline and the biological relevance of the findings. Careful sample selection and dataset-specific comparisons enabled the identification of novel insights across NMDs. In all datasets except ALS_fib_FUS and DMD_cfib, we recovered expected processes such as morphogenesis, synaptic regulation, muscle integrity, inflammation, and extracellular matrix organization [43,44,45,46,47,48]. In DMD_cfib, unlike Soussi et al. [49], we did not detect glycolysis or mitochondrial dysfunction but found structural organization functions. Similarly, in ALS_fib_FUS, our findings contrasted with those of Kumbier et al. [31], who reported enrichment in metabolism, gene expression, and antigen processing. Instead, we detected functions such as cardiac tissue development, kidney morphogenesis, and blood circulation regulation, also present in other differentiated tissue datasets (Supplementary Figure S7). These differences likely reflect divergent methodological aims, as Kumbier et al. focused on subtle progression-related biomarkers through machine learning [31], while our approach emphasized broader transcriptional patterns.

### 3.2. Comparative Functional Enrichment Extends Original DEG Findings

The comparative functional enrichment analysis of DEGs across the different NMD datasets revealed a complex and interconnected landscape of biological functions (Figure 2). While each dataset reflects specific disease contexts, our results show an overlap in functional categories, indicating shared molecular mechanisms that transcend clinical and genetic heterogeneity. Among the most recurrently enriched functions were extracellular matrix binding and glycosaminoglycan binding, present across most datasets. These terms suggest a conserved dysregulation of extracellular matrix (ECM) interactions across NMDs, which may contribute to impaired tissue integrity and aberrant cell signaling in both myogenic and neurogenic contexts [50,51,52]. A noticeable results is the frequent occurrence of the glycosaminoglycan binding term in most DMD-related datasets and in the ALS_fib_FUS dataset. Glycosaminoglycans are vital ECM components that regulate growth factor signaling, neuronal growth, synaptic plasticity, and tissue organization, dysregulation of which has been previously associated with muscular dystrophies like DMD [53,54]. In the context of ALS, and particularly *FUS*-associated ALS, recent studies have emphasized the role of ECM alterations and synaptic dysfunction mediated by RNA-binding proteins like *FUS* [55]. The enrichment of glycosaminoglycan binding shown in the ALS_fib_FUS dataset could suggest that ECM–protein interactions may be implicated in the downstream effects of *FUS* mutations, potentially influencing synaptic integrity and motor neuron survival. And the convergence of this function between DMD and ALS_fib_FUS datasets, despite their genetic heterogeneity, could reflect the potential involvement of ECM dysregulation as a shared pathological mechanism across these diseases. In addition, muscle-related functions, such as actin binding and structural constituent of muscle, were shared specifically between DMD and LGMD datasets derived from cardiomyocytes and myoblasts (DMD_pCard and LGMD_myob, respectively), underscoring common disruptions in cytoskeletal and contractile protein networks. These findings align with the known involvement of cytoskeletal integrity in muscle pathophysiology and suggest that even when different genes are causative, downstream effects on actin filament organization and contractility are preserved across muscular dystrophies [56]. Interestingly, functions such as integrin binding, growth factor binding, and collagen binding were found in DMD- and LGMD-related datasets of similar tissue origins, indicating that tissue context strongly influences which shared pathways are activated or disrupted. Moreover, the detection of peptidase regulator activity as a shared functional category between LGMD_pbmc and ALS_iN_C9ORF72, despite their distinct pathological phenotypes, raises the possibility of convergent regulatory mechanisms involving proteostasis or inflammatory modulation [34].

### 3.3. Insights from Mapping Differential Expression onto the Human Interactome

Despite differences in disease type and cell context across the datasets, the identification of shared genes and conserved biological functions reveals core mechanisms of neuromuscular pathology that are likely of broad significance. This principle drives our integrative systems-level methodology: by mapping differential expression onto the human interactome, we aim to uncover molecular signatures and pathways that consistently emerge across heterogeneous datasets, providing insights not only into disease mechanisms but also into potential therapeutic targets.

Mapping disease-causal genes and their associated DEGs onto the human interactome revealed unexpected patterns of proximity among NMDs, according to results obtained from the dataset view. Notably, despite belonging to clinically distinct diseases, the causal genes *C9ORF72* (ALS) and *TNPO3* (LGMD) are positioned closely in the network and share nearby DEGs primarily from DMD- and LGMD-related datasets. This suggests a potential molecular overlap involving *C9ORF72* and *TNPO3*, despite their association with distinct diseases, and reinforces the idea of a shared pathogenic basis between LGMD and ALS, which may help explain some overlapping molecular features as previously described in the comparative functional enrichment analysis of DEGs. Conversely, *FUS* and *C9ORF72*, both causally linked to ALS, are topologically distant in the interactome, suggesting involvement in distinct molecular mechanisms. The spatial separation observed between *FUS* and its associated DEGs, and similarly between *DMD* and its DEGs, may reflect complex regulatory relationships or tissue-specific expression patterns. These findings demonstrate that, despite the datasets’ limitations, our integrative approach offers new insights into shared pathways and potential therapeutic targets across NMDs.

### 3.4. Biological Relevance of Unmapped DEGs

A notable subset of DEGs across datasets remained unmapped to the human interactome and were classified as unmapped DEGs (uDEGs). Of special interest is *PAX8-AS1*, which is known to participate in transcriptional regulation and chromatin remodeling, with roles in modulating inflammatory and fibrotic responses characteristics of DMD cardiomyopathy [57]. Its over-expression in differentiated tissues and in LGMD_myob may reflect shared pathological mechanisms related to muscle remodeling or stress responses in muscular dystrophies. Given the shared cardiac and fibrotic manifestations in DMD and LGMD [40], this expression pattern raises the possibility that *PAX8-AS1* could serve as a novel biomarker for disease activity or progression in these NMDs. In the case of the *MEG3* gene, it regulates cell differentiation and apoptosis, with roles that vary depending on tissue context [58]. Its over-expression in skeletal muscle cells may reflect a compensatory response to dystrophin deficiency, promoting regenerative or stress-related pathways, while its down-regulation in cardiac cells could indicate a distinct regulatory landscape where *MEG3*-mediated mechanisms are suppressed or differently modulated, possibly contributing to cardiac-specific aspects of DMD [59]. *SBF2-AS1*, an lncRNA involved in regulating cell proliferation, migration, and stress responses [60], shows opposite expression patterns in LGMD myoblasts and ALS iNeurons. Its over-expression in LGMD may reflect a compensatory response to muscle degeneration and inflammation [61], while down-regulation in ALS suggests impaired stress responses linked to *C9ORF72*-related neuronal dysfunction. These tissue-specific differences underscore the role of *SBF2-AS1* as a context-dependent regulatory hub influencing disease progression, making it a promising target to explore shared molecular mechanisms across NMDs. And regarding *NEAT1*, a critical lncRNA for paraspeckle formation and RNA regulation [62], shows contrasting expression in NMDs: It is over-expressed in DMD myoblasts, potentially as an adaptive response to muscle stress and inflammation, helping to maintain transcriptional stability and protect muscle cells. Conversely, its down-regulation in ALS fibroblasts with *FUS* mutations aligns with paraspeckle dysfunction observed in ALS [63], which may impair RNA metabolism and stress responses in neurons. These opposing patterns emphasize tissue-specific paraspeckle roles in disease, and may position *NEAT1* as a promising molecular link and biomarker across muscular dystrophies and neurodegenerative disorders. These findings suggest that lncRNAs may contribute to disease- and tissue-specific regulatory programs in NMDs and represent a layer of molecular complexity not captured by current interactome-based analyses. Nonetheless, these findings should be validated through experimental approaches.

### 3.5. Integrating Differential Expression Profiles with the Human Interactome to Elucidate Disease Mechanisms

The spatial proximity of DEG clusters to causal genes in our integrative network analysis suggests functional associations relevant to disease pathogenesis. In the DMD_myot dataset, the proximity of a DEG cluster enriched in cardiac electrophysiology and muscle contraction functions to the causal gene *DMD* underscores the systemic impact of dystrophin deficiency on both skeletal and cardiac muscle, including disrupted Purkinje fiber signaling and actin-mediated contraction [40,64]. In DMD_pCard, cluster 8 was enriched in processes related to coagulation and hemostasis, supporting the involvement of vascular dysregulation in cardiac manifestations of DMD. Similarly, in DMD_cfib, although fewer DEGs were detected, cluster 0 showed enrichment in cytoskeletal organization and extracellular matrix anchoring, functions directly tied to the role of dystrophin in cardiac fibroblasts and indicative of remodeling processes in the DMD heart. Cluster 7 in the DMD_myob dataset was enriched in genes involved in tissue architecture, extracellular proteolysis, and dystroglycan and apolipoprotein binding, reflecting the impact of dystrophin loss on membrane dynamics, lipid metabolism, and inflammatory remodeling during myogenesis. In the ALS_iN_C9ORF72 dataset, cluster 9 DEGs were enriched in functions related to neuronal homeostasis, including autophagy, synaptic regulation, and mitochondrial maintenance, processes central to C9ORF72-related ALS. Lastly, ALS_fib_FUS cluster 8 showed enrichment in connective tissue development and Wnt signaling regulation, indicating potential systemic and developmental effects of *FUS* mutations in non-neuronal cells [65]. The identified gene clusters and their associated biological processes represent plausible candidates for involvement in disease mechanisms; however, experimental validation is required to confirm their functional relevance.

### 3.6. Biological Relevance and Interpretation of Isolated DEGs

The analysis of isolated DEGs provides insights into specialized or context-specific roles not captured by co-regulated modules. These genes may function in parallel or non-canonical pathways, act in distinct tissues or disease stages, or reflect dynamic regulatory events not apparent in the current dataset. We identified *HS3ST3A1* as a high-scoring, isolated DEG in both LGMD_myob and ALS_fib_FUS, suggesting a previously unrecognized shared role in LGMD and ALS despite lacking direct interaction with *TNPO3* or *FUS*. Similarly, *CDH12* emerged as a consistently isolated DEG in DMD_pCard and DMD_cfib, supporting its involvement in fibroblast-mediated cardiac remodeling in DMD [66] and pointing it out as a potential therapeutic target. Several isolated DEGs in the DMD datasets are biologically relevant despite not clustering. In DMD_myob, *EPSTI1* is linked to interferon signaling and tissue remodeling, suggesting a role in inflammatory responses [67]. In DMD_pCard, *ABCB11* may affect cardiac energetics via lipid transport under stress [68], and in DMD_cfib, *PTPRN* may modulate intercellular communication. These DEGs, although isolated, are functionally proximal to *DMD* and may contribute to tissue-specific manifestations or disease progression. Notably, *DMD* itself was significantly down-regulated in DMD_myot, consistent with previous studies [48] and supporting the robustness of our approach. In the LGMD-related datasets, isolated DEGs found in LGMD_myob, such as *ST6GAL1*, *SRPK3*, and *P2RX5*, may reflect downstream effects of *TNPO3* dysfunction, impacting glycosylation, splicing, and membrane signaling in myoblasts. In LGMD_pbmc, *CHERP* and *EPHA1* suggest systemic effects involving splicing and immune signaling, reinforcing the broad functional impact of *TNPO3* [43]. For the ALS-related datasets, in ALS_iN_C9ORF72, isolated DEGs like *NREP* and *MAP1LC3C* are associated with neuronal regeneration, autophagy, and microtubule dynamics, processes disrupted in ALS [30,69]. Their proximity to *C9ORF72* supports their potential contribution to neurodegeneration [70]. In ALS_fib_FUS, *JMJD6* (involved in chromatin remodeling) and *VRK1* (linked to motor neuron degeneration) both show strong proximity to *FUS*, suggesting convergence on transcriptional and epigenetic dysregulation in ALS pathogenesis [71,72].

### 3.7. Study Limitations

The datasets analyzed in this study encompass different neuromuscular disease contexts (DMD, LGMD, and ALS) and diverse cell types, including myoblasts, myotubes, and fibroblasts, among others. While this heterogeneity introduces variability, it also enables the identification of molecular mechanisms that are consistently deregulated across distinct disease models. Using our integrative approach, we identified convergent alterations, such as shared DEGs and common functional categories, that show potential core disease mechanisms transcending individual cell types or experimental conditions. We note, however, that cell-type-specific conclusions cannot be directly drawn from this analysis, and the findings should be interpreted as reflecting broad, conserved pathways underlying NMDs.

Another limitation consists of the small sample size per dataset, common in rare disease research, which limits statistical power and may hide subtle but biologically relevant expression changes. Access to patient-derived RNA-seq data is further constrained by ethical and legal restrictions, impeding broader data integration. Our network-based framework is also constrained by the incompleteness of current interaction databases, particularly regarding poorly annotated genes and ncRNAs. This gap can lead to the exclusion of potentially important regulators from clustering and prioritization analyses. Future efforts in expanding and curating interaction datasets, especially for lncRNAs, are essential to better capture the regulatory landscape of NMD. Additionally, variability in RNA-seq library preparation protocols across datasets introduces technical noise. PolyA-selected libraries enrich for mature protein-coding RNAs but miss non-polyadenylated transcripts, while ribodepletion includes a broader range of RNAs at the cost of sequencing depth per transcript. These protocol differences should be carefully considered in cross-study analyses and indicate the need for methodological harmonization. Expanding this approach to additional LGMD and ALS subtypes, as more datasets become available (e.g., via SRA), will help refine disease-specific versus shared molecular signatures. Moreover, future versions of our pipeline will incorporate regulatory gene–lncRNA relationships, enabling deeper insight into the non-coding architecture of NMDs. Altogether, our findings generate testable hypotheses on shared disease mechanisms and identify candidate genes for further functional validation.

## 4. Materials and Methods

We selected RNA-seq datasets of two neuromuscular disorders (NMDs) with clinically similar phenotypes [28], Duchenne muscular dystrophy (DMD), caused by mutations in *DMD* [73], and limb–girdle muscular dystrophy type D2 (also known as LGMD *TNPO3*-related or LGMDD2), a rare condition caused by mutations in the *TNPO3* gene [28], to explore shared molecular signatures. Additionally, we included two datasets from amyotrophic lateral sclerosis (ALS), a clinically distinct NMD, each involving different causal genes, to assess disease-specific and convergent mechanisms across diverse pathological contexts.

### 4.1. Dataset Description

We conducted three independent advanced searches in the NCBI Sequence Read Archive (SRA) online platform (https://www.ncbi.nlm.nih.gov/sra), accessed on 20 May 2025, to identify RNA sequencing (RNA-seq) datasets for DMD, LGMD, and ALS. Search queries included the terms “Duchenne Muscular Dystrophy”, “Limb-Girdle Muscular Dystrophy”, and “Amyotrophic Lateral Sclerosis”, restricting results to the *Homo sapiens* organism, “transcriptomic” (RNA) as the source, “Illumina” as the sequence platform, and “fastq” as the file type. Retrieved datasets were manually curated by selecting only those with at least three samples per condition, excluding studies that did not meet this minimum requirement. Additionally, we prioritized datasets generated using paired-end sequencing over single-end reads. Following this selection process, eight datasets were chosen for analysis: four corresponding to DMD with mutations in the *DMD* gene; two for LGMDD2 with mutations in *TNPO3*; and two for ALS, one involving mutations in *C9ORF72* and the other in *FUS* genes.

To facilitate dataset tracking, we assigned standardized names based on disease and cell type and, in the case of ALS datasets, causal gene studied: DMD_myot, DMD_pCard, DMD_cfib, DMD_myob, LGMD_myob, LGMD_pbmc, ALS_iN_C9ORF72, and ALS_fib_FUS. These datasets encompass different Illumina sequencing platforms and sequencing layouts (single-end and paired-end). Additionally, we ensured that samples were obtained from different tissues, including, in the case of DMD, myotubes derived from reprogrammed skin fibroblasts (DMD_myot dataset), cardiomyocytes differentiated from iPSCs reprogrammed from peripheral blood mononuclear cells (PBMCs) in the DMD_pCard dataset, and human-induced pluripotent stem cell (hiPSC) lines derived from cardiac fibroblasts (DMD_cfib dataset) and myoblasts (DMD_myob dataset); in the case of LGMD, primary myoblasts (LGMD_myob dataset) and PBMCs (LGMD_pbmc dataset); and in the case of ALS, induced neurons (iNeurons) for the ALS_iN_C9ORF72 dataset and fibroblasts for the ALS_fib_FUS dataset. More information on these datasets is provided in Table 3.

It is important to note that in our study, the differential expression analyses focused not on the specific location of the mutations within the genes analyzed, but rather on the broader impact of these genetic alterations on the expression of other genes. Furthermore, we carefully selected samples for each dataset, prioritizing controls from healthy individuals over isogenic controls, as they better reflect differential expression between healthy and diseased conditions. When isogenic controls were used, we chose those generated with the most effective gene-editing techniques to minimize potential biases in our results. It is also important to consider that our integrative analysis methodology is specifically designed with the limitations of rare disease research in mind, particularly the typically low number of samples available per dataset. Further details of the selected RNA-seq datasets are included in Supplementary Table S3.

For the DMD_myot dataset, Paredes-Redondo et al. [48] investigated neuromuscular junction defects and their potential impact on DMD pathogenesis. DMD patient fibroblasts carrying the c.10141C>T (p.R3381X) nonsense mutation, which affects all tissue-specific dystrophin isoforms, were reprogrammed into expanded potential stem cells, which were differentiated into embryoid bodies, and an isogenic control line was generated by correcting the mutation via CRISPR-Cas9. To determine whether the impaired differentiation and fusion observed in DMD-cells stemmed from abnormal myogenic gene expression, they conducted transcriptome sequencing and analysis of DMD patient-derived myotubes and the corrected DMD myogenic cultures at 0, 24, and 120 h during secondary differentiation. To capture more mature transcriptional profiles that better reflect the functional consequences of the DMD mutation, we selected samples belonging to 120 h of differentiation. A total of three samples per condition (CRISPR/Cas9 isogenic-corrected controls and DMD patient samples) were analyzed.

Regarding the DMD_pCard dataset, Atmanli et al. [44] investigated the structural, functional, and transcriptional differences in cardiomyocytes derived from iPSCs reprogrammed from PBMS of a DMD patient. The original study comprised nine samples: three from the patient and six from CRISPR/Cas9 isogenic-corrected controls in which the *DMD* open reading frame was restored either by reframing (three samples) or exon skipping (three samples). For our comparison, we selected samples corrected by reframing, as this approach aims to preserve the near-full-length protein structure, potentially leading to a more physiologically accurate rescue of the dystrophin gene [74].

In the case of the DMD_cfib dataset, Soussi et al. [49] analyzed the gene expression profiles from hiPSC-derived cardiac fibroblasts obtained from DMD patients and healthy controls. DMD samples lack the full-length dystrophin isoform due to gene mutations, leading to impaired actin microfilament organization and a metabolic shift from oxidative phosphorylation to glycolysis. These cells also show disrupted mitochondrial networks, reduced mitochondrial respiration, and an enhanced myofibroblast phenotype in response to profibrotic stimuli. As cardiac fibrosis is a hallmark of DMD-related cardiomyopathy, this model provides insights into how dystrophin deficiency in non-cardiomyocyte cells contributes to disease progression. We used the eight samples available at the SRA, with four corresponding to control individuals and four corresponding to patients with mutations in the *DMD* gene.

Regarding the latest DMD dataset (DMD_myob), Lemoine et al. [46] carried out a study demonstrating the effectiveness of a single guide RNA CRISPR strategy to delete exon duplications in the DMD gene (exon 2, exons 2–9, and exons 8–9) in patient-derived myogenic cells, demonstrating that correction restored dystrophin expression and normalized related gene pathways, as shown by immunostaining and RNA-seq. In our study, we did not investigate the effects of CRISPR correction. Instead, we focused on performing differential expression analysis using samples from patients with different DMD mutations and a healthy control. Regarding this consideration, we analyzed the samples corresponding to myoblasts from patients carrying exon 2, exon 2–9, and exon 8–9 duplications in the DMD gene, along with samples from an immortalized myoblasts cell line (C25) belonging to a healthy individual. Each condition was represented by three replicates.

For the case of LGMD, we selected two datasets corresponding to LGMDD2, the *TNPO3*-related subtype of LGMD. For clarification, throughout the manuscript we refer to this disease subtype as LGMD. For the first dataset, LGMD_myob, published by Poyatos-Garcia et al. [47], they used a patient-derived immortalized myoblast model that recapitulates disease features, including *TNPO3* over-expression, impaired muscle differentiation, and autophagy dysregulation. CRISPR-Cas9 correction of the mutation reversed these phenotypes, eliminating the mutant protein and restoring 44% of transcriptomic alterations and 50% of dysregulated miRNAs. Muscle biopsies were obtained from the tibialis anterior of a 33-year-old male patient diagnosed with LGMD and from the quadriceps of a 38-year-old healthy male donor. Primary human myoblasts derived from these biopsies were immortalized through lentiviral transduction with hTERT and CDK4 vectors, followed by clonal selection. For each condition (LGMD and healthy control), three independent clonal lines were established and used as biological replicates. These immortalized myoblasts were cultured under standard growth conditions and subsequently differentiated to analyze disease-associated molecular alterations. In our study, we compared the three samples belonging to the healthy control against the LGMD patient samples.

In the second LGMD dataset (LGMD_pbmc) Diez-Fuertes et al. [43] explored the molecular basis of LGMD, performing a transcriptome analysis of PBMCs from LGMD patients with the c.2771delA mutation in the *TNPO3* gene against healthy individuals, revealing differentially expressed genes (DEGs) with pro-inflammatory and antiviral process functions associated. This dataset includes 20 samples: 10 from LGMD patients with the deletion c.2771delA in the *TNPO3* gene and 10 samples from healthy individuals.

For the ALS_iN_C9ORF72 dataset, Castelli et al. [45] investigated the effects of neuroprotection in induced neurons (iNeurons) derived from ALS patients carrying expansion mutations in the *C9ORF72* gene. The study focused on a gene therapy approach aimed at inhibiting the SRSF1-dependent nuclear export of C9ORF72 repeat transcripts. For our analysis, we first selected 12 human samples from induced neurons (iNeurons) derived from three healthy individuals and three C9ORF72-ALS patients from whole-cell (WCT) and cytoplasmatic (CyT) transcriptomes. However, we realized that for the ALS_iN_C9ORF72 dataset, the samples belonging to the control (Ctrl_R2_WCT) and patient (ALS_R2_WCT) R2, in the whole-cell transcriptome (WCT), presented a high percentage of reads with incomplete alignments (Supplementary Figure S1G), and, consequently, both were discarded from the analysis. Therefore, due to the limited number of samples in each group, which would not lead to a robust differential expression analysis (even though this comparison would have been the most consistent with the rest of this study), we decided to analyze the comparison between control and ALS patient samples from the cytoplasmic transcriptome (CyT) instead.

And regarding the ALS_fib_FUS dataset, Kumbier et al. [31] investigated whether fibroblasts derived from ALS patients could be used to identify phenotypic heterogeneity in both sporadic ALS and FUS-mutated ALS cases. From the original dataset, we selected 25 samples, comprising 13 controls and 12 FUS-mutated ALS samples.

### 4.2. Dataset Processing for Differential Expression Analysis

All FASTQ files from the datasets were processed using the analysis methodology previously described in [75]. It performs a first quality analysis over raw sequence files using FastQC and then a pre-processing of the reads using SeqtrimBB, an in-house tool built on the BBmap framework [76]. The minimum Phred quality per nucleotide for all datasets was set to 26. The pre-processed read files were aligned against the human reference genome (version GRCh38.p13) using STAR [77], and the count tables obtained for each sample were aggregated into a single file of counts per gene and sample to analyze differential gene expression with the R/Bioconductor package ExpHunter Suite [78].

To ensure consistent and biologically meaningful detection of gene expression across datasets, we applied a filtering threshold of at least two counts per million (CPM) in a minimum number of libraries, adapted proportionally to the sample size of each comparison. For instance, in comparisons with smaller groups (e.g., three vs. three), genes were retained if they reached this expression level in at least two libraries per group. In larger datasets, the threshold was scaled accordingly, requiring expression in at least two-thirds of the libraries per group. This proportional filtering strategy helped minimize noise from lowly expressed or sporadic transcripts, ensuring a more reliable identification of DEGs while maintaining comparability across datasets of varying sizes. All differential expression analyses were conducted using DESeq2 [79], as implemented in the ExpHunter Suite [78], and log_2_FC and *p*-value information is available in Table 3. We selected different log_2_FC cutoffs depending on the number of DEGs obtained for the comparisons performed for each dataset, with the aim of balancing sensitivity and specificity to detect relevant genes and minimize noise, respectively.

Genes that passed the filtering criteria and were identified by DESeq2 were considered DEGs and selected for functional analysis. Functional enrichment was performed using the ExpHunter Suite module based on clusterProfiler [80], with annotations from Gene Ontology (GO) [81]. The over-representation analysis (ORA) method was applied, evaluating both over-expressed and down-regulated DEGs through a hypergeometric test for each term in the selected functional categories. An adjusted *p*-value threshold of 0.05, using the Benjamini–Hochberg (BH) method, was used across all datasets.

### 4.3. Integrative Analysis

To perform the integrative analysis, we constructed a protein–protein interaction (PPI) network using data from the “Experimental” channel of the STRING database (v12.0 [82], https://string-db.org (accessed on 26 May 2025)), which compiles physical interactions supported by laboratory evidence. The distribution of the confidence score for this STRING channel is displayed in Supplementary Figure S10. To ensure reliability, we retained only interactions with a confidence score above 150. ENSEMBL protein identifiers were mapped to their corresponding gene identifiers, and we kept all the genes that were found to be expressed in at least one of the eight datasets. Additionally, a connected component filter was applied, removing all nodes that belonged to connected components (subsets of nodes in which every node is reachable from any other) with fewer than five nodes. Then, DEGs from each study were projected onto the interactome. The resulting DEG subgraphs were clustered, and for each study, we computed the proximity between each cluster and the corresponding causal gene (*DMD*, *TNPO3*, *C9ORF72*, and *FUS*) to identify biological pathways and potential therapeutic targets. All network analyses were conducted using NetAnalyzer [83], a Python library developed by our group (v1.0, https://pypi.org/project/NetAnalyzer (accessed on 27 May 2025)). The complete analysis workflow is available at https://github.com/lhurtadogarcia/degs2net (accessed on 10 July 2025), and the interactive reports for all datasets analyzed are available in the integrative analysis results repository at https://github.com/ElenaRojano/INTRINSED_datasets (accessed on 29 July 2025).

The PPI network derived from each dataset was clustered using the Louvain method [84], following previous studies [85], and implemented through the main module of NetAnalyzer [86]. To ensure structural coherence and biological relevance, we kept clusters containing three or more nodes. Then, the proximity between each resulting cluster and the corresponding causal gene was computed. To achieve this, a similarity matrix was constructed by applying the *node2vec* algorithm [87] with parameters dimensions = 128, walk_length = 100, num_walks = 10, p = 1, q = 1, window = 10 to the degree-normalized adjacency matrix of the network. The resulting similarity matrix represents the dot product of node vectors embedded in a high-dimensional space. To account for differences in vector magnitudes, we normalized these values using the cosine similarity metric. With this matrix, the *ranker* module of NetAnalyzer was used to estimate the average proximity between all genes in each cluster and the causal gene.

Rather than relying solely on the arithmetic mean of proximities, we defined average proximity as the probability that a causal gene is functionally associated with the genes within a given cluster. To estimate this probability, we used a logistic regression model trained on the similarity scores to predict the likelihood that two genes were originally connected in the PPI network. For each gene within a cluster, the model provided a probability of association with the causal gene. Next, we aggregated these probabilities for all genes within the cluster using Fisher’s combined probability test, which allows for robust prioritization of candidate genes. The resulting score value is then rank-normalized based on the distribution of proximities for all genes in the network. In this framework, clusters with a higher average proximity to the causal gene were those in which the causal gene had the highest rank in terms of proximity.

In addition to the quantitative network analyses, an exploratory visual analysis was conducted to facilitate the interpretation and navigation of the PPI network. For this purpose, we generated a representation of the identified clusters using *net_explorer*, a visualization module included in the NetAnalyzer library. This tool offers both an interactive network graph visualized using the Sigma.js v2 JavaScript library, with 200 layout iterations to optimize the positioning of nodes, and a UMAP-based dimensionality reduction plot, enabling intuitive exploration of gene–gene relationships in a two-dimensional space.

For the Uniform Manifold Approximation and Projection (UMAP) visualization, node embeddings were first computed using the *node2vec* algorithm (with parameters dimensions = 64, walk_length = 30, num_walks = 200, p = 1, q = 1, window = 10). These embeddings provided the coordinates for each node, which were then used as input for UMAP projection [88] (n_neighbors = 15, min_dist = 0.1, n_components = 2, metric = ‘euclidean’). This method allowed us to create a simplified, yet informative, representation of the gene interaction network, making it easier to explore patterns within gene clusters and to generate hypotheses about which genes are functionally close to the disease-causing genes.

**Figure 6:**
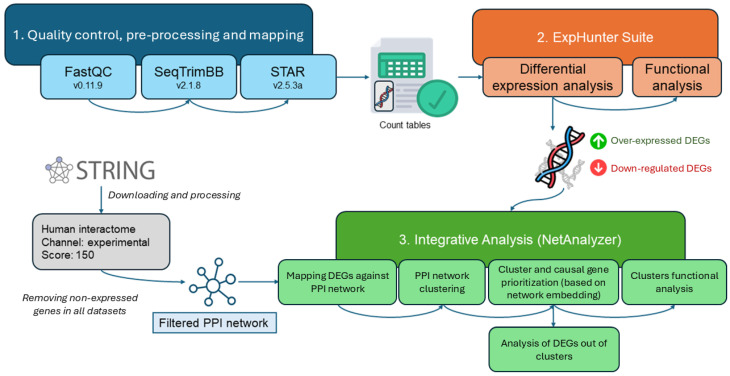
Workflow overview of the analysis pipeline. (1.) RNA-seq data undergo quality control, trimming, and alignment to the hg38 reference genome. (2.) ExpHunter Suite is used to identify differentially expressed genes (DEGs) and perform functional enrichment analysis. (3.) An integrative network analysis is conducted using a filtered STRING interactome built from the union of expressed genes across all datasets, allowing the exploration of DEG clustering around each disease-causal gene.

Finally, a functional analysis of the clusters was performed using *clusterProfiler* with GO biological process annotations. A threshold of 0.05 was applied to the *p*-values, which were adjusted for multiple testing using the BH method. Figure 6 illustrates a conceptual scheme of the methodology developed for this study.

## 5. Conclusions

Our integrative approach uncovers biologically and clinically relevant patterns across diverse neuromuscular diseases (NMDs), revealing mechanisms not detectable by differential expression alone. Comparative functional enrichment analysis of DEGs across all datasets revealed shared functions among several diseases, including glycosaminoglycan binding enrichment in both DMD and *FUS*-related ALS, suggesting that extracellular matrix–protein interactions may contribute to the downstream effects of *FUS* mutations. By combining transcriptomic data with protein–protein interaction networks and clustering based on proximity to causal genes, we identified shared and specific modules enriched in immune response, RNA metabolism, and cytoskeletal organization. Recurrently dysregulated lncRNAs, such as *PAX8-AS1*, *SBF2-AS1*, and *NEAT1*, indicate the regulatory roles of ncRNAs and their potential as cross-disease biomarkers. The prioritization of genes like *HS3ST3A1*, located near both *TNPO3* and *FUS*, illustrates how network context can reveal hidden connections between genetically distinct disorders. Our framework is applicable to other rare and common diseases, offering a scalable tool for uncovering molecular mechanisms and identifying novel therapeutic targets through systems-level analysis.

## Figures and Tables

**Table 1:** Summary of expressed and differentially expressed genes (DEGs) across datasets, including counts of over-expressed and down-regulated genes. DMD: Duchenne muscular dystrophy; LGMD: limb–girdle muscular dystrophy; ALS: amyotrophic lateral sclerosis.

Dataset	Expressed Genes	DEGs	Over-Expressed	Down-Regulated
DMD_myot	13,085	176	137	39
DMD_pCard	12,832	437	38	399
DMD_cfib	13,157	80	30	50
DMD_myob	14,466	580	356	224
LGMD_myob	13,838	548	199	349
LGMD_pbmc	13,598	160	108	52
ALS_iN_C9ORF72	13,453	325	284	41
ALS_fib_FUS	13,004	171	96	75

**Table 2:** Summary of the integrative analysis results across datasets. It includes the number of differentially expressed genes (DEGs) that mapped against the interaction network (Mapped DEGs), the number of DEGs that were left out of the network of interactions, referred to as unmapped differentially expressed genes (uDEGs), the number of coding uDEGs (Coding uDEGs), non-coding RNAs (ncRNAs), and pseudogenes (Pseudo), and the number of DEG clusters. DMD: Duchenne muscular dystrophy; LGMD: limb–girdle muscular dystrophy; ALS: amyotrophic lateral sclerosis.

Dataset	Mapped DEGs	uDEGs	Unmap. Coding	ncRNA	Pseudo	Clusters
DMD_myot	34	17	2	13	2	8
DMD_pCard	247	31	6	22	3	17
DMD_cfib	13	14	2	9	3	4
DMD_myob	239	164	14	114	30	13
LGMD_myob	206	120	11	85	20	15
LGMD_pbmc	19	53	12	19	19	4
ALS_iN_C9ORF72	127	50	3	40	2	10
ALS_fib_FUS	21	36	5	25	3	4

**Table 3:** Summary of datasets used in the study, including the name of the dataset, number of samples per dataset, causal gene, average read size (ARS) per dataset, minimum read length (MRL) set to trim reads, ExpHunter Suite parameters, including minimum libraries selected (MLS) and log_2_ fold change (log_2_FC), and reference of the samples’ original studies. ALS: amyotrophic lateral sclerosis, DMD: Duchenne muscular dystrophy, LGMD: limb–girdle muscular dystrophy.

Dataset	Samples	Causal Gene	ARS	MRL	MLS	log_2_FC	Ref.
DMD_myot	6 (3 isogenic controls + 3 DMD)	*DMD*	75	60	2	1.5	[48]
DMD_pCard	6 (3 CRISPR-Cas9 corrected + 3 DMD)	*DMD*	74	60	2	1.5	[44]
DMD_cfib	8 (4 healthy controls + 4 DMD)	*DMD*	100	85	2	1	[49]
DMD_myob	12 (3 controls + 9 patients)	*DMD*	150	135	2	1.5	[46]
LGMD_myob	6 (3 controls + 3 patients)	*TNPO3*	150	135	2	2.5	[47]
LGMD_pbmc	20 (10 controls and 10 patients)	*TNPO3*	76	61	2	1	[43]
ALS_iN_C9ORF72	12 (6 controls + 6 C9ORF72-ALS)	*C9ORF72*	150	135	2	0.6	[45]
ALS_fib_FUS	25 (13 controls + 12 FUS-ALS)	*FUS*	150	135	8	0.6	[31]

## Data Availability

The Gene Expression Omnibus (GEO) codes for each dataset employed in this study and their references are as follows: GSE159273, (DMD_myot) [48]; GSE169190, (DMD_pCard) [44]; GSE237014, (DMD_cfib) [49]; GSE272233, (DMD_myob) [46]; GSE198551, (LGMD_myob) [47]; GSE193662, (LGMD_pbmc) [43]; GSE139900, (ALS_iN_C9ORF72) [45]; GSE234991, (ALS_fib_FUS) [31].

## Supplementary Materials

The following supporting information can be downloaded at https://www.mdpi.com/article/10.3390/ijms26199376/s1.

## Abbreviations

The following abbreviations are used in this manuscript:

ALS	Amyotrophic Lateral Sclerosis
ARS	Average Read Size
BH	Benjamini–Hochberg
CPM	Counts Per Million
CyT	Cytoplasmatic Transcriptome
DEG	Differentially Expressed Gene
DMD	Duchenne Muscular Dystrophy
ECM	Extracellular Matrix
GEO	Gene Expression Omnibus
GO	Gene Ontology
HCPC	Hierarchical Clustering on Principal Components
hiPSC	Human-Induced Pluripotent Stem Cell
iPSCs	Induced Pluripotent Stem Cells
LGMD	Limb–Girdle Muscular Dystrophy
LGMDD2	Limb–Girdle Muscular Dystrophy D2
lncRNAs	Long Non-Coding RNAs
MLS	Minimum Libraries Selected
MRL	Minimum Read Length
NCBI	National Center for Biotechnology Information
NMDs	Neuromuscular Diseases
ORA	Over-Representation Analysis
PBMCs	Peripheral Blood Mononuclear Cells
PCA	Principal Component Analysis
PPI	Protein–Protein Interaction
RNA-seq	RNA Sequencing
SRA	Sequence Read Archive
UMAP	Uniform Manifold Approximation and Projection
WCT	Whole-Cell Transcriptome
