# Supplementary material for: Integrative Transcriptomic and Network-Based Analysis of Neuromuscular Diseases
Source: Int J Mol Sci. 2025 Sep 25;26(19):9376. doi: 10.3390/ijms26199376 (PMC12525345; doi:10.3390/ijms26199376)
Supplement: Supplementary file 1 [file ijms-26-09376-s001.zip › ijms-3817812-supplementary.pdf]

Supplementary material

Table S1. Top ten differentially expressed genes (DEGs) ranked by their log_2FC (from highest to lowest) for each dataset DMD dataset (DMD_myot, DMD_pCard, DMD_cfib and DMD_myob).

ID	log_2FC	Gene Symbol	Dataset
ENSG00000214548	9.329897687	*MEG3*	DMD_myot
ENSG00000287080	8.680853606	*H3C3*	DMD_myot
ENSG00000155008	8.501931016	*APOOL*	DMD_myot
ENSG00000225746	8.46109789	*ENSG00000225746*	DMD_myot
ENSG00000162623	7.769389631	*TYW3*	DMD_myot
ENSG00000171094	-3.492998891	*ALK*	DMD_myot
ENSG00000114654	-3.042854954	*EFCC1*	DMD_myot
ENSG00000170290	-2.810693768	*SLN*	DMD_myot
ENSG00000133020	-2.565352591	*MYH8*	DMD_myot
ENSG00000120057	-2.432159111	*SFRP5*	DMD_myot
ENSG00000189223	5.88822191	*PAX8-AS1*	DMD_pcard
ENSG00000159248	5.434954133	*GJD2*	DMD_pcard
ENSG00000171864	3.421100698	*PRND*	DMD_pcard
ENSG00000110799	3.14559456	*VWF*	DMD_pcard
ENSG00000048540	2.849823199	*LMO3*	DMD_pcard
ENSG00000124107	-8.794610564	*SLPI*	DMD_pcard
ENSG00000077274	-8.58463897	*CAPN6*	DMD_pcard
ENSG00000124205	-8.001103953	*EDN3*	DMD_pcard
ENSG00000155011	-7.868336554	*DKK2*	DMD_pcard
ENSG00000196090	-7.819216639	*PTPRT*	DMD_pcard
ENSG00000169436	9.024092122	*COL22A1*	DMD_cfib
ENSG00000189223	6.566983729	*PAX8-AS1*	DMD_cfib
ENSG00000082482	5.883638995	*KCNK2*	DMD_cfib
ENSG00000144834	5.868433019	*TAGLN3*	DMD_cfib
ENSG00000171502	4.995137047	*COL24A1*	DMD_cfib
ENSG00000012504	-7.474132943	*NR1H4*	DMD_cfib
ENSG00000234444	-6.104334548	*ZNF736*	DMD_cfib
ENSG00000106483	-5.865551797	*SFRP4*	DMD_cfib
ENSG00000081842	-5.178089797	*PCDHA6*	DMD_cfib
ENSG00000224549	-5.022565823	*ENSG00000224549*	DMD_cfib
ENSG00000214548	8.564267813	*MEG3*	DMD_myob
ENSG00000134184	7.908815517	*GSTM1*	DMD_myob
ENSG00000225746	7.744089875	*ENSG00000225746*	DMD_myob
ENSG00000187688	7.686184497	*TRPV2*	DMD_myob
ENSG00000175161	7.531063627	*CADM2*	DMD_myob
ENSG00000174469	-7.7155905	*CNTNAP2*	DMD_myob
ENSG00000157601	-6.814552143	*MX1*	DMD_myob
ENSG00000228318	-6.809012077	*ENSG00000228318*	DMD_myob
ENSG00000184486	-6.625125528	*POU3F2*	DMD_myob
ENSG00000130303	-6.312210882	*BST2*	DMD_myob

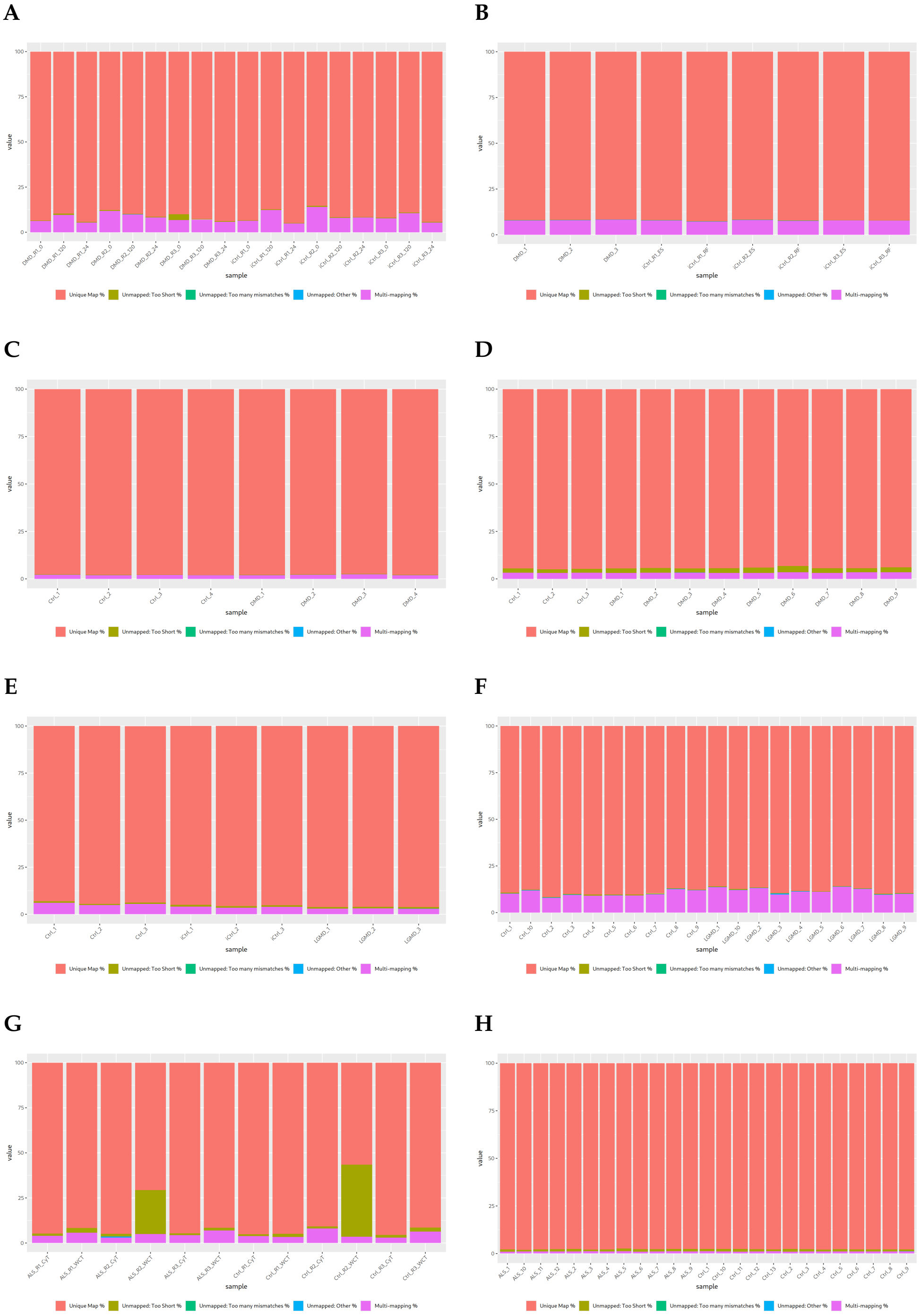

Figure S1. Distribution of the percentage of reads and how they mapped against the reference genome using STAR. Datasets: (A) DMD_myot, (B) DMD_pCard, (C) DMD_cfib, (D) DMD_myob, (E) LGMD_myob, (F) LGMD_pbmc, (G) ALS_iN_C9ORF72 and (H) ALS_fib_FUS. For each sample, the percentage of uniquely mapped reads (red bars), unmapped reads, including too short (olive green), many mismatches (green) and others (blue), along with multimapping reads (pink) is represented.

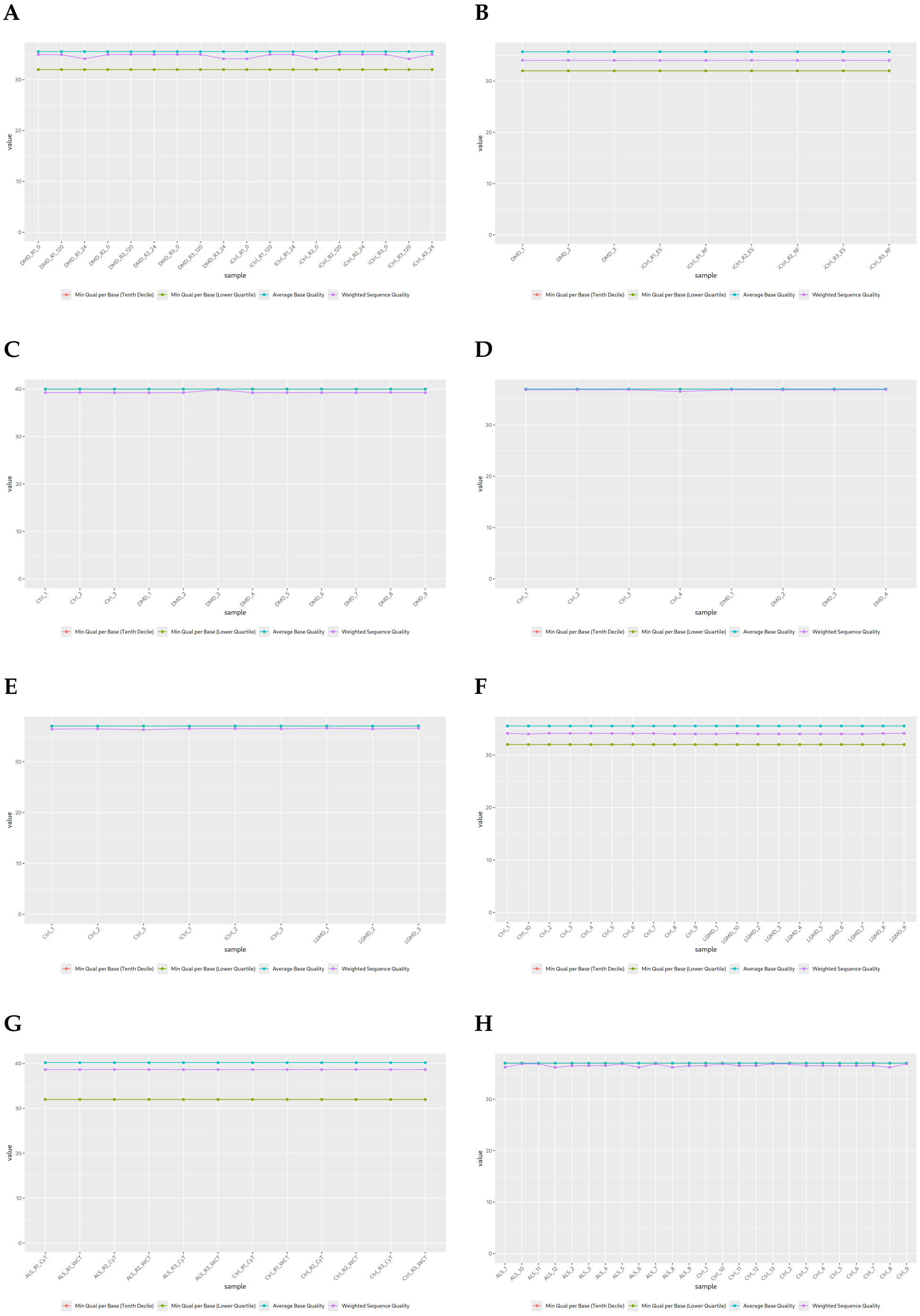

Figure S2. Quality scores after pre-processing samples with SeqTrimBB. Information is retrieved from FastQC. The minimum QUAL per base in the tenth decile (red), lower quartile (green), average base quality (light blue) and weighted sequence quality (mean Phred score across its bases) is included. Datasets: (A) DMD_myot, (B) DMD_pCard, (C) DMD_cfib, (D) DMD_myob, (E) LGMD_myob, (F) LGMD_pbmc, (G) ALS_iN_C9ORF72 and (H) ALS_fib_FUS.

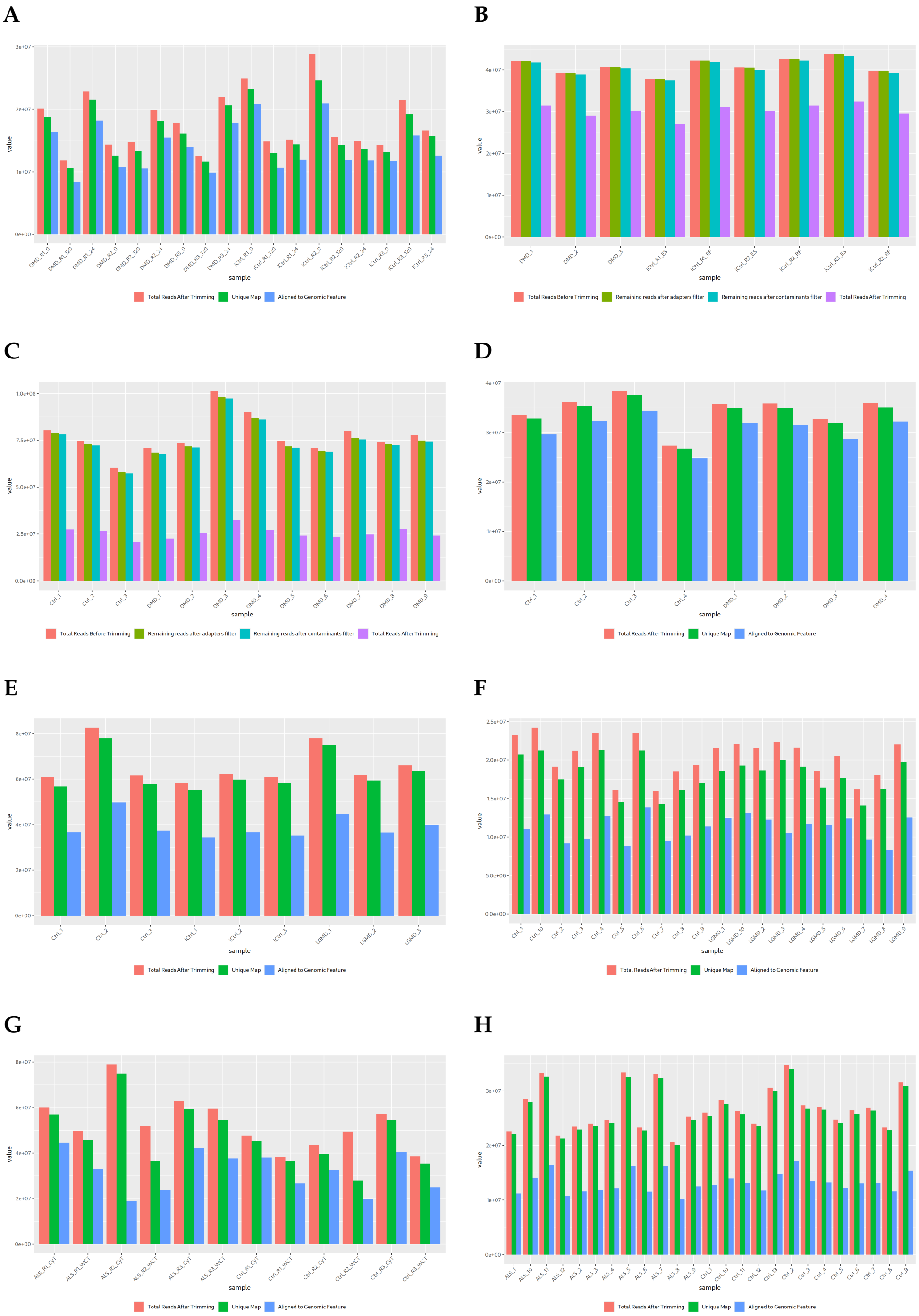

Figure S3. Summary of the mapping statistics for each dataset. For every dataset included in the study, the bar chart displays the total number of reads remaining after the pre-processing step (red bars), the number of reads that aligned uniquely to the reference genome (green bars), and, among these, the subset that mapped to annotated genes (blue bars). Datasets: (A) DMD_myot, (B) DMD_pCard,(C) DMD_cfib, (D) DMD_myob, (E) LGMD_myob, (F) LGMD_pbmc, (G) ALS_iN_C9ORF72 and (H) ALS_fib_FUS.

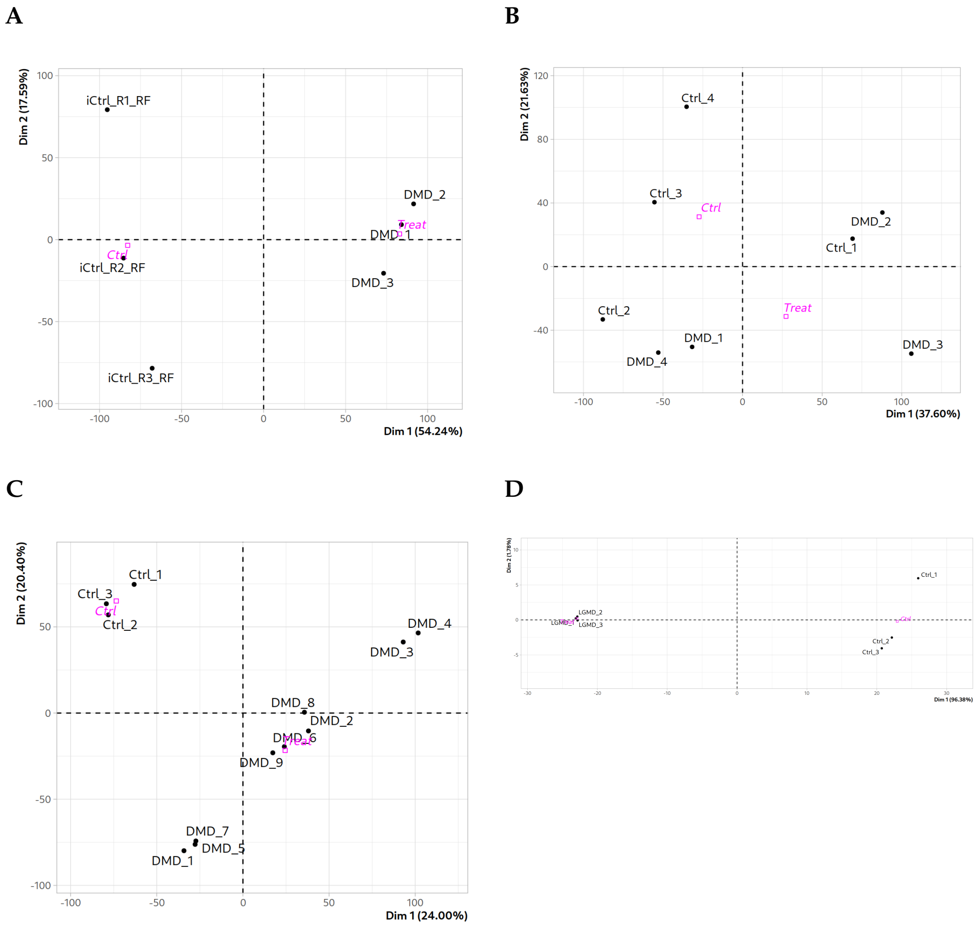

Figure S4. Principal component analysis (PCA) performed for the following datasets: A) DMD_pCard, B) DMD_cfib, C) DMD_myob and D) LGMD_myob. The centroid of each group of samples compared (Ctrl and Treat) is displayed. DMD: Duchenne muscular dystrophy, LGMD: Limb-girdle muscular dystrophy.

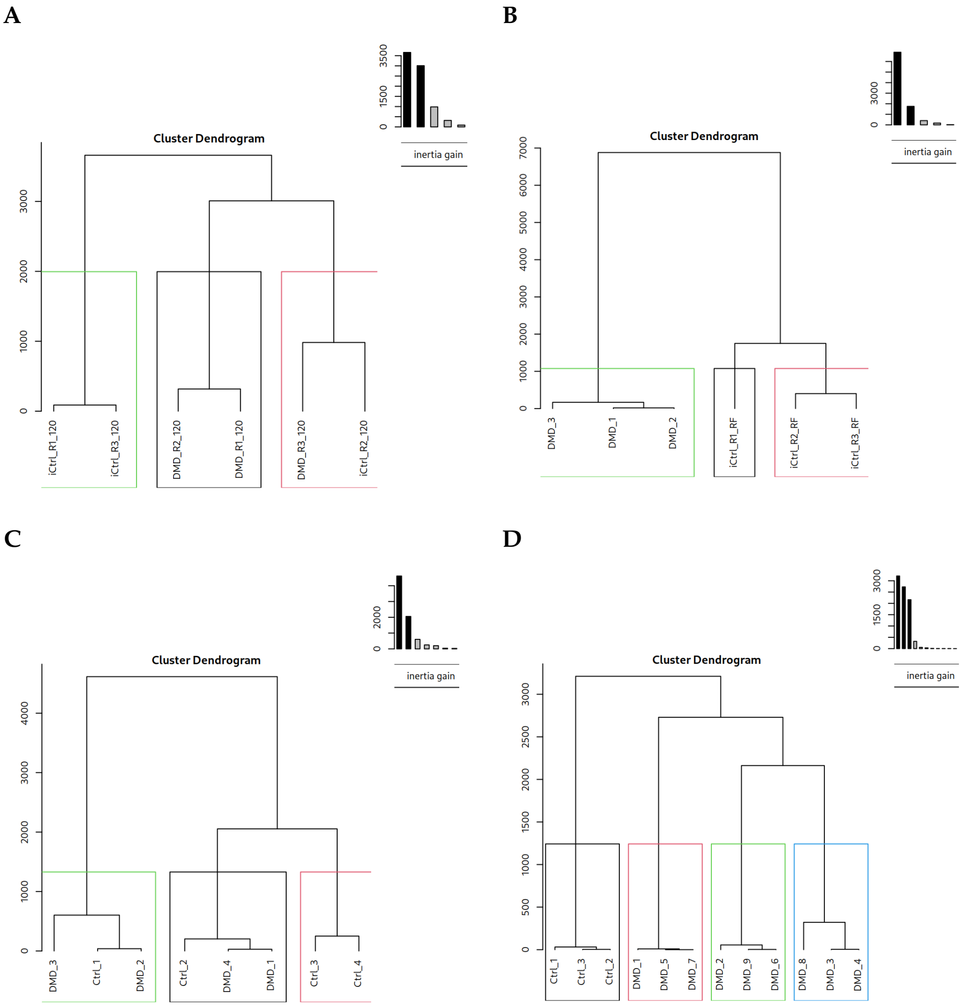

Figure S5. Hierarchical Clustering on Principal Components (HCPC) performed over the different datasets for DMD datasets: DMD_myot (A), DMD_pCard (B), DMD_cfib (C), DMD_myob (D). Distance is displayed in the Y-axis. Inertia gain is visualized as a bar graph where each bar represents the increase in inertia at a potential cut point in the dendrogram; individual branching events are explicitly shown, and the optimal cut is determined at the branching point with the highest inertia gain.

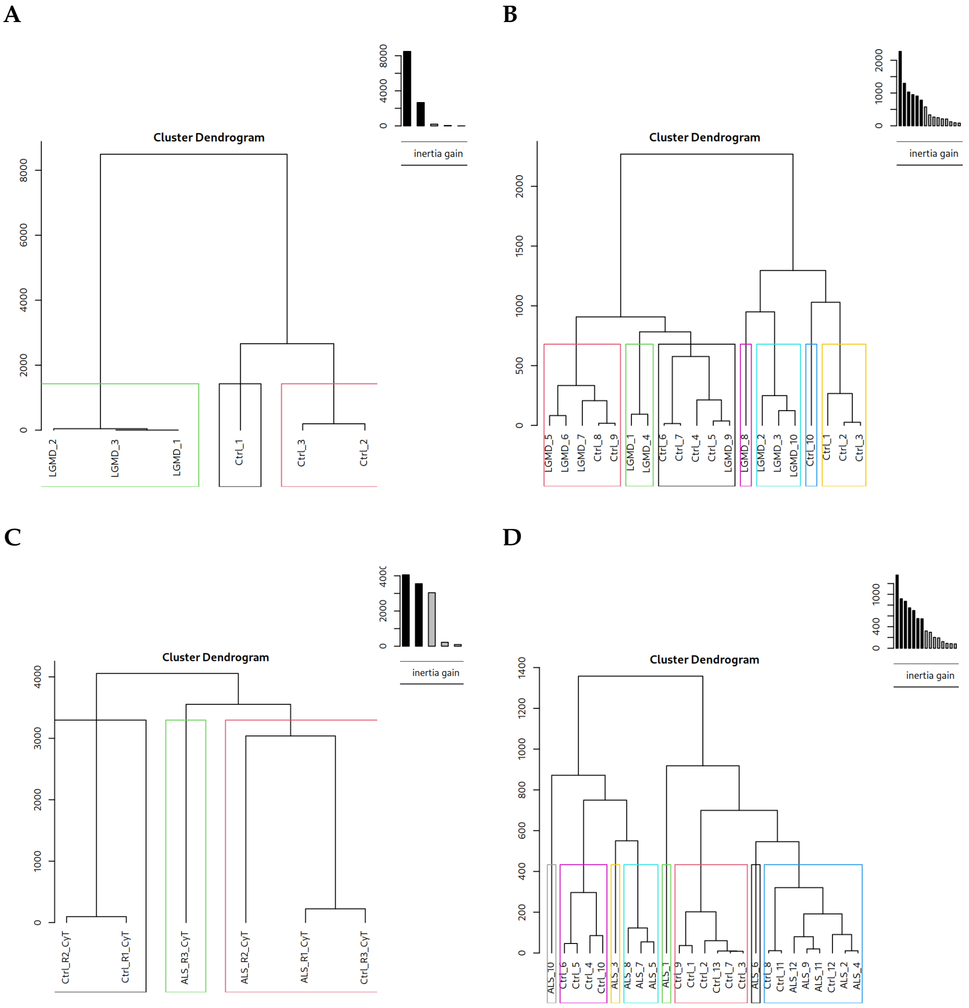

Figure S6. Hierarchical Clustering on Principal Components (HCPC) performed over the differ-ent datasets for LGMD datasets: LGMD_myob (A) and LGMD_pbmc (B), and for ALS datasets: ALS_iN_C9ORF72 (C), ALS_fib_FUS (D). Distance is displayed in the Y-axis. Inertia gain is visual-ized as a bar graph where each bar represents the increase in inertia at a potential cut point in the dendrogram; individual branching events are explicitly shown, and the optimal cut is determined at the branching point with the highest inertia gain.

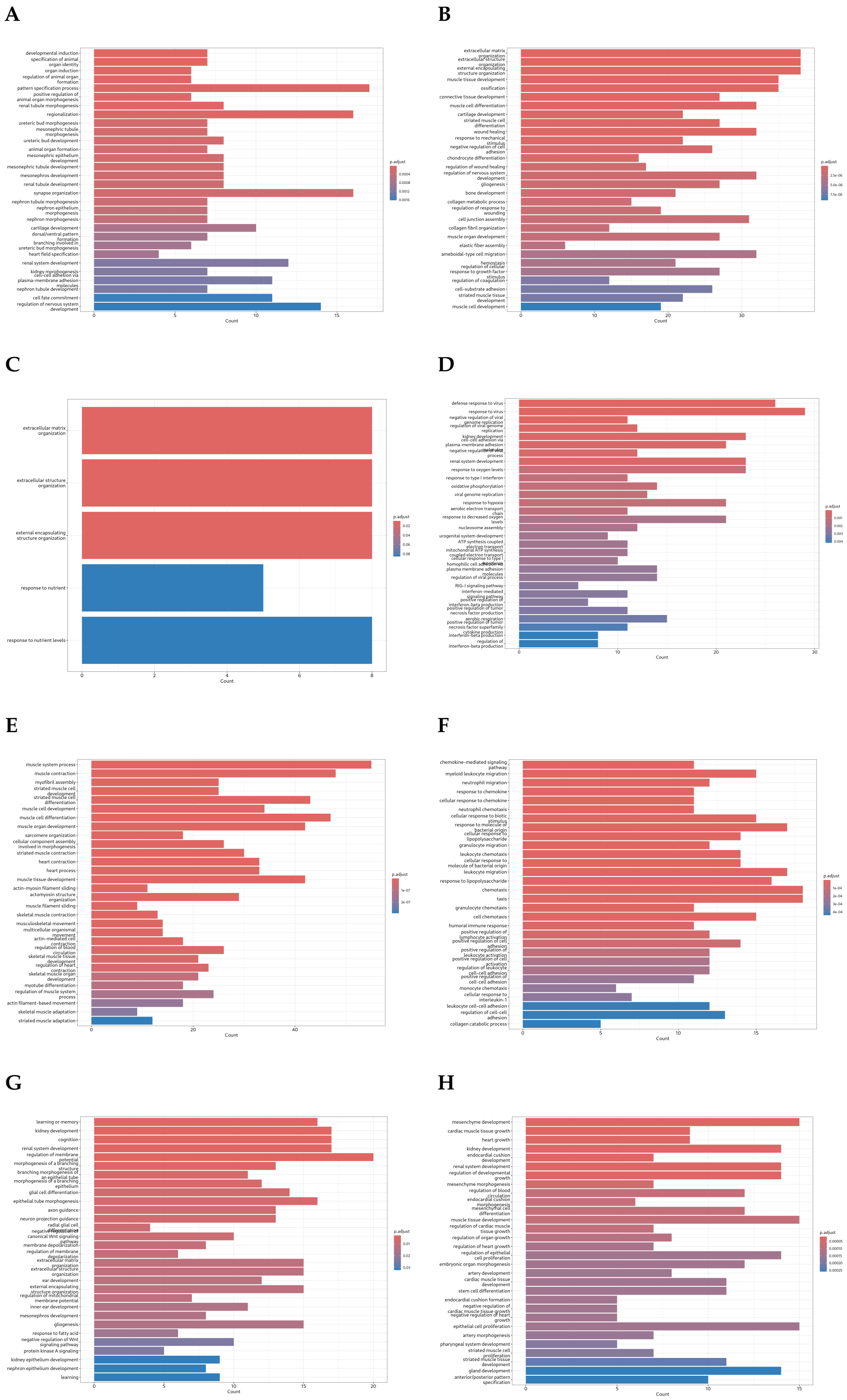

Figure S7. Barplots from the Over Representation Analysis (ORA) performed over differential ex-pressed genes (DEGs) from the different datasets: (A) DMD_myot, (B) DMD_pcard, (C) DMD_cfib,(D) DMD_myob, (E) LGMD_myob, (F) LGMD_pbmc, (G) ALS_iN_C9ORF72 and (H) ALS_fib_FUS. Categories shown belong to the Gene Ontology biological process.

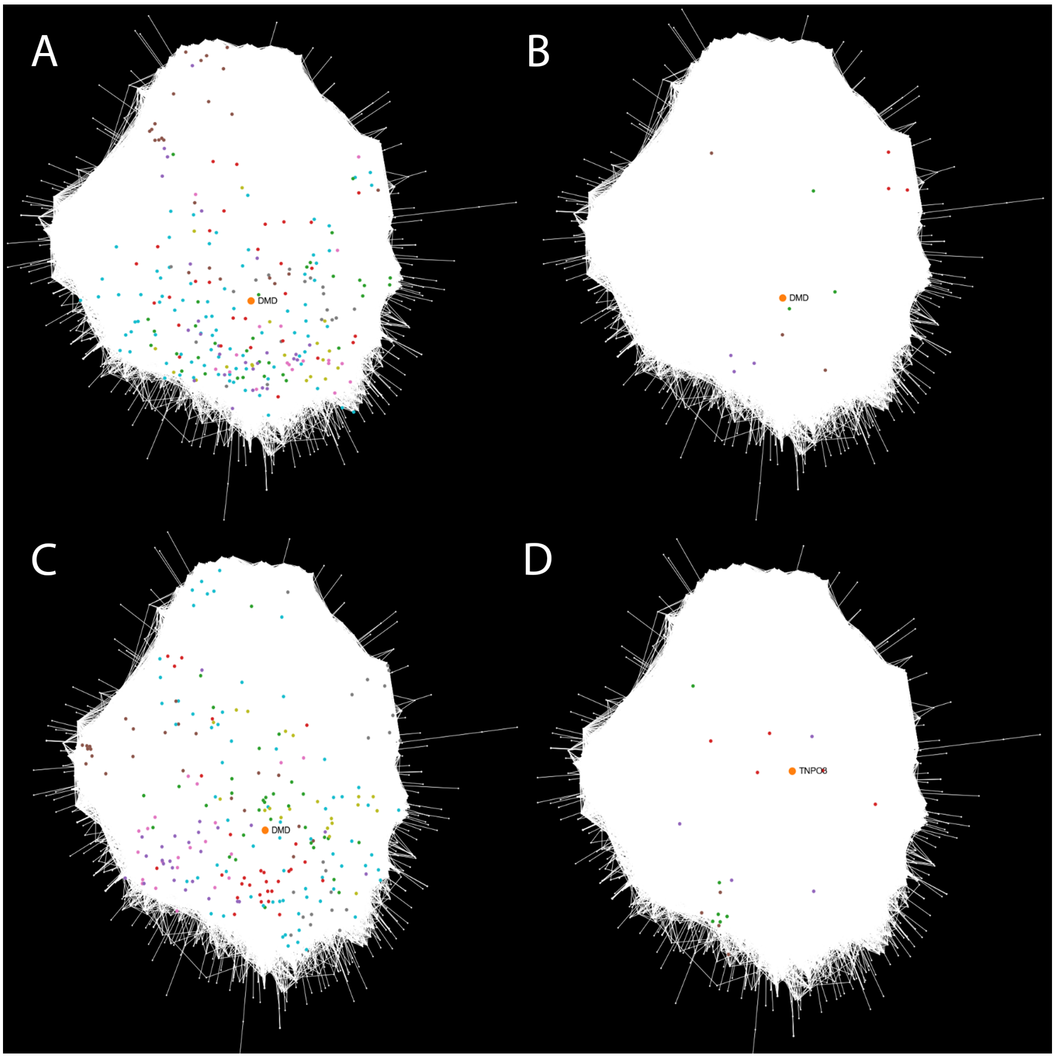

Figure S8. Protein-protein interaction networks based on the union of all expressed genes across datasets. For each dataset, the DEG clusters are shown in different colors, revealing their clustering patterns. The orange dot indicates the disease-related gene used as a reference (*DMD* and *TNPO3*). A) DMD_pCard, B) DMD_cfib, C) DMD_myob and D) LGMD_pbmc datasets.

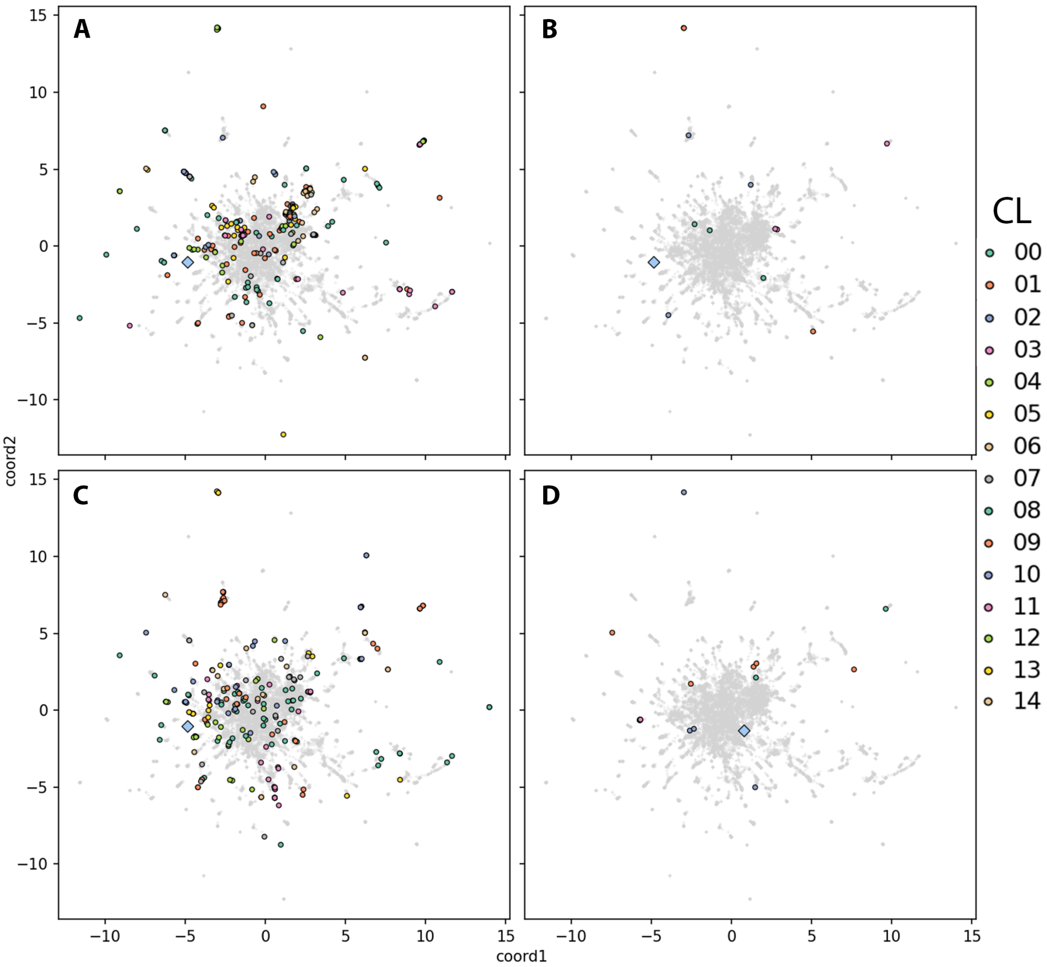

Figure S9. Two-dimensional embedding of gene interaction networks. Each panel represents the reduced vector space (coord1 vs. coord2) of the full gene interaction network based on STRING "Experimental" evidence. Background grey dots correspond to all expressed genes included in the network. The disease-causal gene (*seed*) for each condition (*DMD*, *TNPO3*, *C9ORF72* and *FUS*) is represented as a blue rhombus. Panels correspond to the DMD_pCard (A), DMD_cfib (B), DMD_myob (C) and LGMD_pbmc (D) datasets. Colored dots represent differentially expressed genes (DEGs), grouped by cluster (CL).

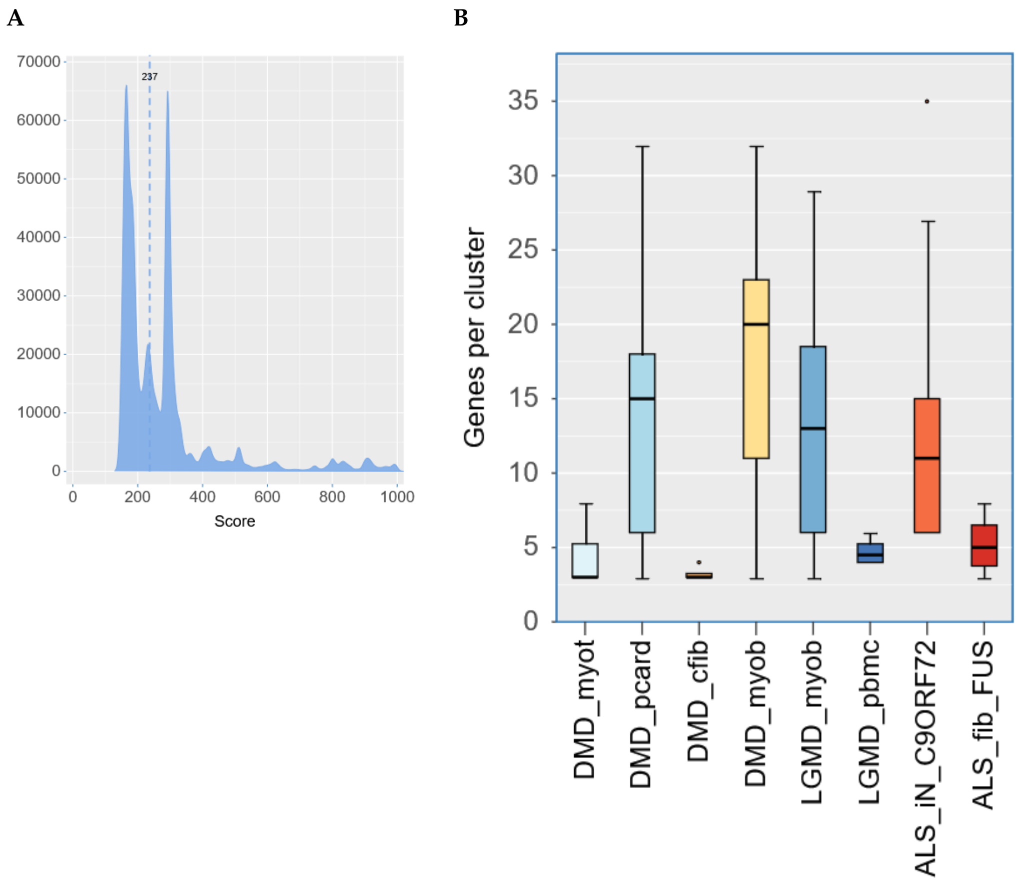

Figure S10. A) STRING "Experimental" channel scores distribution. B) Distribution of DEGs per cluster in all datasets (DMD_myot, DMD_pcard, DMD_cfib, DMD_myob, LGMD_myob, LGMD_pbmc, ALS_iN_C9ORF72 and ALS_fib_FUS).

Table S2. Top ten differentially expressed genes (DEGs) ranked by their $\log_2$FC (from highest to lowest) for each LGMD dataset (LGMD_myob and LGMD_pbmc) and for each ALS dataset (ALS_iN_C9ORF72 and ALS_fib_FUS datasets.)

ID	$\log_2$FC	Gene Symbol	Dataset
ENSG00000183549	9.76967333	*ACSM5*	LGMD_myob
ENSG00000275895	9.549062963	*LOC102724594*	LGMD_myob
ENSG00000228340	9.474992105	*MIR646HG*	LGMD_myob
ENSG00000183801	9.268052923	*OLFML1*	LGMD_myob
ENSG00000134184	9.166709346	*GSTM1*	LGMD_myob
ENSG00000225630	-9.348707076	*MTND2P28*	LGMD_myob
ENSG00000187688	-9.107161129	*TRPV2*	LGMD_myob
ENSG00000179455	-9.061608271	*MKRN3*	LGMD_myob
ENSG00000241945	-8.990805562	*PWP2*	LGMD_myob
ENSG00000275620	-8.720037735	*FLJ16779*	LGMD_myob
ENSG00000196611	6.246250316	*MMP1*	LGMD_PBMC
ENSG00000197632	6.072554488	*SERPINB2*	LGMD_PBMC
ENSG00000163735	5.765536582	*CXCL5*	LGMD_PBMC
ENSG00000074410	5.268491204	*CA12*	LGMD_PBMC
ENSG00000163734	5.247146242	*CXCL3*	LGMD_PBMC
ENSG00000228078	-5.692259109	*ENSG00000228078*	LGMD_PBMC
ENSG00000152672	-3.462160924	*CLEC4F*	LGMD_PBMC
ENSG00000249917	-2.336057043	*LINC00536*	LGMD_PBMC
ENSG00000278416	-2.152524027	*ENSG00000278416*	LGMD_PBMC
ENSG00000262402	-2.097058208	*ENSG00000262402*	LGMD_PBMC
ENSG00000250366	9.581682298	*TUNAR*	ALS_iN_C9ORF72
ENSG00000181291	9.575731861	*TMEM132E*	ALS_iN_C9ORF72
ENSG00000269994	9.540754174	*LINC02893*	ALS_iN_C9ORF72
ENSG00000204287	9.448847287	*HLA-DRA*	ALS_iN_C9ORF72
ENSG00000162998	9.398092467	*FRZB*	ALS_iN_C9ORF72
ENSG00000175793	-6.11840715	*SFN*	ALS_iN_C9ORF72
ENSG00000148671	-5.095503897	*ADIRF*	ALS_iN_C9ORF72
ENSG00000101210	-5.07538562	*EEF1A2*	ALS_iN_C9ORF72
ENSG00000011590	-4.611922474	*ZBTB32*	ALS_iN_C9ORF72
ENSG00000188042	-4.598501103	*ARL4C*	ALS_iN_C9ORF72
ENSG00000171724	2.199740968	*VAT1L*	ALS_fib_FUS
ENSG00000130720	1.917163957	*FIBCD1*	ALS_fib_FUS
ENSG00000204055	1.737743134	*ENSG00000204055*	ALS_fib_FUS
ENSG00000175084	1.718878774	*DES*	ALS_fib_FUS
ENSG00000117152	1.683603117	*RGS4*	ALS_fib_FUS
ENSG00000115457	-3.606169709	*IGFBP2*	ALS_fib_FUS
ENSG00000164530	-3.479066099	*PI16*	ALS_fib_FUS
ENSG00000106483	-2.811743963	*SFRP4*	ALS_fib_FUS
ENSG00000086289	-2.38666159	*EPDR1*	ALS_fib_FUS
ENSG00000168672	-2.139848876	*LRATD2*	ALS_fib_FUS

Table S3. Summary of the datasets used in this study, including the dataset name, the causative mutation, the time of cell culture differentiation (Time of CC dif.), the platform (Illumina sequencer) and layout selected, the average number of reads per sample (Avg. NRPS), the description of samples and cell type used to perform the differential expression analysis. DMD: Duchenne muscular distrophy. LGMD: limb-girdle muscular distrophy. ALS: amyotrophic lateral scoliosis. PBMC: peripheral blood ononuclear cells. iNPC: induced neural progenitor cells.

**Dataset**	**Causative mutation**	**Time of CC dif.**	**Platform**	**Layout**	**Avg. NRPS**	**Samples description**	**Cell type**
DMD_myot	DMD c.10141C>T	120 hours	NextSeq 500	Paired	1×10^7	9 isogenic controls and 9 DMD samples (three biological replicates of two genotypes at three time points)	Myotubes derived from reprogrammed skin fibroblasts
DMD_pcard	*DMD* ΔEx44	Day 50 post differentiation	NextSeq 500	Single	4×10^7	3 controls and 6 CRISPR-corrected samples (3 by exon-skipping and 3 by reframing)	Cardiomyocytes differentiated from iPSCs reprogrammed from PBMCs
DMD_cfib	DMD ΔEx49,50; ΔEx45,50; c.6913–1G>A	—	NextSeq 500	Paired	3×10^7	4 controls and 4 DMD patients	Human iPSC lines differentiated into cardiac fibroblasts
DMD_myob	Dup2, Dup2-9, Dup8-9	Day 8 of differentiation	NovaSeq 6000	Paired	4×10^7	3 controls and 9 patients (3 with Dup2, 3 with Dup2-9, 3 with Dup8-9)	Myoblasts
LGMD_myob	*TNPO3* c.2771delA	—	NovaSeq 6000	Paired	1.5×10^8	3 controls, 3 isogenic controls and 3 patients	Primary myoblasts
LGMD_pbmc	*TNPO3* c.2771delA	—	NextSeq 550	Single	2×10^7	10 controls and 10 patients from the same family	PBMCs
ALS_iN_C9ORF72	CS28iALS-C9nxx; RRID:CVCL_W558 & CS29iALS-C9nxx; RRID:CVCL_W559	Day 40	HiSeq 4000	Paired	4.5×10^7	6 controls (3 whole-cell transcriptomes, 3 cytoplasmic) and 6 patients	iNPC-derived iNeurons
ALS_fib_FUS	*FUS*(c.1545_1547dup, p.Glu515_Glu516insAsp), g.11194C>G (p.H517Q) - H517Q, FUS-R521G, *FUS*-P525L, FUS-R524W, c.1566G>A (p.R522R) - G522A, 1561C>G (p.R521G), *FUS* R495X	—	HiSeq 4000	Paired	3×10^7	13 controls, 12 FUS-ALS patients, 11 sporadic ALS patients	Fibroblasts

Table S4. Common differentially expressed non-coding RNAs not reported in the STRING interactome network.

Gene Symbol	log_2FC	Datasets
ENSG00000210082	6.66271761, 2.350697846	DMD_myot, DMD_myob
ENSG00000211459	6.856519466, 1.769644484	DMD_myot, DMD_myob
ENSG00000225746	8.46109789, -1.61975765, 7.744089875	DMD_myot, DMD_pcard, DMD_myob
ENSG00000228509	-1.500228158, -4.619953197	DMD_pcard, LGMD_myob
ENSG00000234665	-5.303053232, 6.612476245	LGMD_myob, ALS_iN_C9ORF72
ENSG00000236333	2.583009658, 6.398757626	DMD_myot, ALS_iN_C9ORF72
ENSG00000247134	7.082599117, -3.918320817, 1.052531096	DMD_myob, LGMD_myob, LGMD_pbmc
ENSG00000260604	-1.633556296, 2.770659015	DMD_myob, LGMD_myob
ENSG00000264490	2.620604786, -3.239567081	DMD_myob, LGMD_myob
ENSG00000267328	4.314826556, 0.939302737	DMD_myob, ALS_fib_FUS
ENSG00000268518	1.620199672, -3.410659159	DMD_myob, LGMD_myob
ENSG00000275266	-1.611720439, -3.11208589	DMD_pcard, LGMD_myob
ENSG00000281344	2.259690595, 3.131760569, 3.327599889	DMD_myob, LGMD_myob, ALS_iN_C9ORF72
H19	-1.671813204, -2.741273729	DMD_pcard, LGMD_myob
LINC01615	1.585841416, 3.337558246	DMD_myob, LGMD_myob
MEG3	9.329897687, -2.168895462, 8.564267813	DMD_myot, DMD_pcard, DMD_myob
MIR1915HG	-2.303961547, 1.167736333	DMD_cfib, ALS_fib_FUS
MIR206	1.777347446, -4.167276676	DMD_myob, LGMD_myob
MIR381HG	6.155384396, -2.648835477	DMD_myot, DMD_pcard
NEAT1	2.156031047, -1.464471	DMD_myob, ALS_fib_FUS
PAX8-AS1	4.242991409, 5.88822191, 6.566983729, -4.979454426, 4.695141887	DMD_myot, DMD_pcard, DMD_cfib, DMD_myob, LGMD_myob
PCOLCE-AS1	4.098649958, 2.550229585	DMD_myob, LGMD_myob
SBF2-AS1	3.71442084, -2.264803297	LGMD_myob, ALS_iN_C9ORF72
SCAT8	-1.550985117, -4.568172847	DMD_myob, LGMD_myob
SNHG18	5.533150502, -7.97648756	DMD_myob, LGMD_myob

Table S5. Clustered DEGs priorization results for the datasets DMD_myot, DMD_pcard, DMD_cfib and DMD_myot. Here we show the disease-causal gene identifiers (ENSG00000198947: *DMD*), their corresponding clustering scores (score), normalized ranks (Norm. rank), and raw ranks (considering the total expressed genes in the network), alongside their assigned causal gene group and correspond-ing dataset.

Causal gene	Score	Norm. rank	Rank	Group	Dataset
ENSG00000198947	0.77	0.02	400	3	DMD_myot
ENSG00000198947	0.04	0.35	5244	7	DMD_myot
ENSG00000198947	0.03	0.34	4986	6	DMD_myot
ENSG00000198947	0.02	0.26	3791	2	DMD_myot
ENSG00000198947	9.87e-04	0.78	11476	5	DMD_myot
ENSG00000198947	6.96e-04	0.81	11860	4	DMD_myot
ENSG00000198947	4.97e-04	0.59	8747	1	DMD_myot
ENSG00000198947	7.55e-05	0.54	7983	0	DMD_myot
ENSG00000198947	1.00	0.00	37	8	DMD_pcard
ENSG00000198947	0.05	0.32	4758	15	DMD_pcard
ENSG00000198947	0.03	0.37	5430	16	DMD_pcard
ENSG00000198947	0.01	0.38	5613	12	DMD_pcard
ENSG00000198947	0.00	0.27	4058	10	DMD_pcard
ENSG00000198947	0.00	0.64	9357	14	DMD_pcard
ENSG00000198947	0.00	0.46	6770	13	DMD_pcard
ENSG00000198947	4.87e-04	0.59	8705	11	DMD_pcard
ENSG00000198947	9.35e-06	0.34	4984	5	DMD_pcard
ENSG00000198947	9.15e-06	0.32	4696	6	DMD_pcard
ENSG00000198947	7.06e-06	0.36	5255	4	DMD_pcard
ENSG00000198947	8.29e-07	0.31	4611	3	DMD_pcard
ENSG00000198947	4.74e-08	0.44	6518	2	DMD_pcard
ENSG00000198947	2.32e-08	0.69	10176	7	DMD_pcard
ENSG00000198947	3.98e-10	0.85	12401	9	DMD_pcard
ENSG00000198947	4.14e-12	0.56	8210	1	DMD_pcard
ENSG00000198947	1.24e-15	0.73	10740	0	DMD_pcard
ENSG00000198947	1.00	0.00	3	0	DMD_cfib
ENSG00000198947	0.03	0.34	5220	1	DMD_cfib
ENSG00000198947	1.41e-03	0.81	12689	2	DMD_cfib
ENSG00000198947	0.02	0.42	6480	3	DMD_cfib
ENSG00000198947	4.65e-07	0.21	3245	0	DMD_myob
ENSG00000198947	2.43e-22	0.97	15047	1	DMD_myob
ENSG00000198947	1.07e-08	0.41	6386	2	DMD_myob
ENSG00000198947	1.34e-11	0.61	9445	3	DMD_myob
ENSG00000198947	1.03e-13	0.86	13395	4	DMD_myob
ENSG00000198947	4.05e-05	0.22	3410	5	DMD_myob
ENSG00000198947	1.30e-08	0.51	7919	6	DMD_myob
ENSG00000198947	0.94	0.00	41	7	DMD_myob
ENSG00000198947	7.75e-16	0.97	15066	8	DMD_myob
ENSG00000198947	1.09e-04	0.47	7334	9	DMD_myob
ENSG00000198947	0.11	0.10	1592	10	DMD_myob
ENSG00000198947	2.78e-03	0.32	4947	11	DMD_myob
ENSG00000198947	0.02	0.42	6468	12	DMD_myob

Table S6. Clustered DEGs priorization results for the datasets LGMD_myob, LGMD_pbmc, ALS_iN_C9ORF72 and ALS_fib_FUS. Here we show the disease-causal gene identifiers (ENSG00000064419: *TNPO3*, ENSG00000147894: *C9ORF72* and ENSG00000089280: *FUS*), their corresponding clustering scores (score), normalized ranks (Norm. rank), and raw ranks (considering the total expressed genes in the network), alongside their assigned causal gene group and correspond-ing dataset.

Causal gene	Score	Norm. rank	Rank	Group	Dataset
ENSG00000064419	5.42E-16	0.75	11605	0	LGMD_myob
ENSG00000064419	1.15E-10	0.51	7974	1	LGMD_myob
ENSG00000064419	2.07E-10	0.43	6714	2	LGMD_myob
ENSG00000064419	1.03E-09	0.68	10657	3	LGMD_myob
ENSG00000064419	8.48E-10	0.74	11528	4	LGMD_myob
ENSG00000064419	1.89E-06	0.51	7897	5	LGMD_myob
ENSG00000064419	9.25E-14	0.94	14600	6	LGMD_myob
ENSG00000064419	1.59E-05	0.36	5627	7	LGMD_myob
ENSG00000064419	8.72E-03	0.17	2644	8	LGMD_myob
ENSG00000064419	2.85E-06	0.82	12801	9	LGMD_myob
ENSG00000064419	1.09E-09	1.00	15528	10	LGMD_myob
ENSG00000064419	9.90E-04	0.59	9141	11	LGMD_myob
ENSG00000064419	1.53E-02	0.36	5546	12	LGMD_myob
ENSG00000064419	1.96E-04	0.75	11756	13	LGMD_myob
ENSG00000064419	9.73E-03	0.48	7452	14	LGMD_myob
ENSG00000064419	5.21E-07	0.89	13904	0	LGMD_pbmc
ENSG00000064419	2.78E-04	0.73	11417	1	LGMD_pbmc
ENSG00000064419	5.99E-02	0.21	3212	2	LGMD_pbmc
ENSG00000064419	1.05E-03	0.65	10148	3	LGMD_pbmc
ENSG00000147894	0.31	0.08	1231	9	ALS_iN_C9ORF72
ENSG00000147894	0.11	0.06	928	3	ALS_iN_C9ORF72
ENSG00000147894	4.18e-04	0.58	8573	7	ALS_iN_C9ORF72
ENSG00000147894	5.21e-05	0.73	10767	8	ALS_iN_C9ORF72
ENSG00000147894	1.83e-05	0.72	10614	4	ALS_iN_C9ORF72
ENSG00000147894	3.68e-06	0.83	12243	5	ALS_iN_C9ORF72
ENSG00000147894	1.43e-06	0.96	14067	6	ALS_iN_C9ORF72
ENSG00000147894	2.23e-07	0.43	6279	2	ALS_iN_C9ORF72
ENSG00000147894	1.58e-13	0.88	12956	1	ALS_iN_C9ORF72
ENSG00000147894	9.59e-23	0.91	13329	0	ALS_iN_C9ORF72
ENSG00000089280	0.02	0.35	5118	2	ALS_fib_FUS
ENSG00000089280	0.01	0.54	7901	3	ALS_fib_FUS
ENSG00000089280	4.07e-04	0.64	9366	1	ALS_fib_FUS
ENSG00000089280	2.35e-04	0.45	6648	0	ALS_fib_FUS

Table S7. Gene Ontology (GO) biological process enrichment results for cluster (CL) 3 in the DMD_myot dataset. Metrics include GeneRatio (GR), background ratio (BgRatio), RichFactor, Fold Enrichment, z-score, and adjusted p-value.

CL	ID	Description	GR	BgRatio	RichFactor	FoldEnrichment	zScore	p.adjust	geneID	Count
3	GO:0098911	regulation of ventricular cardiac muscle cell action potential	0.67	12/18888	0.17	1049.33	45.78	0.00007	*PKP2/DSC2*	2
3	GO:0086069	bundle of His cell to Purkinje myocyte communication	0.67	15/18888	0.13	839.47	40.94	0.00007	*PKP2/DSC2*	2
3	GO:0098901	regulation of cardiac muscle cell action potential	0.67	29/18888	0.07	434.21	29.43	0.00016	*PKP2/DSC2*	2
3	GO:0086004	regulation of cardiac muscle cell contraction	0.67	34/18888	0.06	370.35	27.17	0.00016	*PKP2/DSC2*	2
3	GO:0086005	ventricular cardiac muscle cell action potential	0.67	35/18888	0.06	359.77	26.78	0.00016	*PKP2/DSC2*	2
3	GO:0070252	actin-mediated cell contraction	0.67	113/18888	0.02	111.43	14.84	0.00047	*PKP2/DSC2*	2
3	GO:0030048	actin filament-based movement	0.67	138/18888	0.01	91.25	13.41	0.00066	*PKP2/DSC2*	2
3	GO:0006936	muscle contraction	0.67	355/18888	0.01	35.47	8.26	0.00283	*PKP2/DSC2*	2

Table S8. Gene Ontology (GO) biological process enrichment results for cluster (CL) 0 in the DMD_cfib dataset. Metrics such as GeneRatio (GR), background ratio (BgRatio), RichFactor, Fold Enrichment, z-score, and adjusted p-value are included.

CL	ID	Description	GR	BgRatio	RichFactor	FoldEnrichment	zScore	p.adjust	geneID	Count
0	GO:0002162	dystroglycan binding	0.25	5.94e-4	0.09	4.21e+2	20.48	7.96e-3	*DMD*	1
0	GO:0050998	nitric-oxide synthase binding	0.25	5.94e-4	0.09	4.21e+2	20.48	7.96e-3	*DMD*	1
0	GO:0017166	vinculin binding	0.25	6.48e-4	0.08	3.86e+2	19.60	7.96e-3	*DMD*	1
0	GO:0003779	actin binding	0.50	2.34e-2	0.00	2.13e+1	6.30	7.96e-3	*ANLN/DMD*	2
0	GO:0008307	structural constituent of muscle	0.25	2.27e-3	0.02	1.10e+2	10.42	1.81e-2	*DMD*	1

Table S9. Gene Ontology (GO) biological process enrichment results for cluster (CL) 8 in the DMD_pCard dataset. Displayed metrics include GeneRatio (GR), background ratio (BgRatio), RichFactor, Fold Enrichment, z-score, and adjusted p-value.

CL	ID	Description	GR	BgRatio	RichFactor	FoldEnrichment	zScore	p.adjust	geneID	Count
8	GO:0030195	negative regulation of blood coagulation	0.20	49/18888	0.06	77.09	15.04	0.00157	*PLAT/TFPI/SERPINE2*	3
8	GO:1900047	negative regulation of hemostasis	0.20	50/18888	0.06	75.55	14.88	0.00157	*PLAT/TFPI/SERPINE2*	3
8	GO:0050819	negative regulation of coagulation	0.20	53/18888	0.06	71.28	14.44	0.00157	*PLAT/TFPI/SERPINE2*	3
8	GO:0030193	regulation of blood coagulation	0.20	69/18888	0.04	54.75	12.61	0.00208	*PLAT/TFPI/SERPINE2*	3
8	GO:1900046	regulation of hemostasis	0.20	71/18888	0.04	53.21	12.42	0.00208	*PLAT/TFPI/SERPINE2*	3
8	GO:0060384	innervation	0.07	27/18888	0.04	46.64	6.69	0.09417	*SERPINE2*	1

Table S10. Gene Ontology (GO) biological process enrichment results for cluster (CL) 7 in the DMD_myob dataset. Metrics such as GeneRatio (GR), background ratio (BgRatio), RichFactor, Fold Enrichment, z-score, and adjusted p-value are included.

7	GO:0002162	dystroglycan binding	0.10	5.94e-4	0.18	1.68e+2	18.26	5.88e-3	*AGRN/DMD*	2
7	GO:0034185	apolipoprotein binding	0.10	9.18e-4	0.12	1.09e+2	14.64	7.24e-3	*LRP4/PCSK9*	2
7	GO:0005201	extracellular matrix structural constituent	0.15	8.96e-3	0.02	1.67e+1	6.70	1.86e-2	*AGRN/THBS2/PCOLCE*	3
7	GO:0004252	serine-type endopeptidase activity	0.15	9.40e-3	0.02	1.60e+1	6.52	1.86e-2	*MMP2/PCSK9/FAP*	3
7	GO:0008236	serine-type peptidase activity	0.15	1.03e-2	0.02	1.45e+1	6.19	1.86e-2	*MMP2/PCSK9/FAP*	3

Table S11. Gene Ontology (GO) biological process enrichment results for cluster (CL) 9 in the ALS_iN_C9ORF72 dataset. Metrics such as GeneRatio (GR), background ratio (BgRatio), RichFactor, Fold Enrichment, z-score, and adjusted p-value are included.

CL	ID	Description	GR	BgRatio	RichFactor	FoldEnrichment	zScore	p.adjust	geneID	Count
9	GO:0004252	serine-type endopeptidase activity	0.50	174/18522	0.01	53.22	7.19	0.02793	*RHBDL3*	1
9	GO:0008236	serine-type peptidase activity	0.50	191/18522	0.01	48.49	6.86	0.02793	*RHBDL3*	1
9	GO:0017171	serine hydrolase activity	0.50	195/18522	0.01	47.49	6.78	0.02793	*RHBDL3*	1
9	GO:0004175	endopeptidase activity	0.50	388/18522	0.00	23.87	4.73	0.04146	*RHBDL3*	1

Table S12. Gene Ontology (GO) biological process enrichment results for cluster (CL) 2 in the ALS_fib_FUS dataset. Metrics such as GeneRatio (GR), background ratio (BgRatio), RichFactor, Fold Enrichment, z-score, and adjusted p-value are included.

CL	ID	Description	GR	BgRatio	RichFactor	FoldEnrichment	zScore	p.adjust	geneID	Count
2	GO:0061448	connective tissue development	0.75	288/18888	0.01	49.19	11.99	0.00414	*SLC25A25/AMER1/NOG*	3
2	GO:0060612	adipose tissue development	0.50	59/18888	0.03	160.07	17.81	0.00854	*SLC25A25/AMER1*	2
2	GO:0090090	negative regulation of canonical Wnt signaling pathway	0.50	145/18888	0.01	65.13	11.28	0.03453	*AMER1/NOG*	2
2	GO:0030178	negative regulation of Wnt signaling pathway	0.50	177/18888	0.01	53.36	10.19	0.03817	*AMER1/NOG*	2
2	GO:0060828	regulation of canonical Wnt signaling pathway	0.50	268/18888	0.01	35.24	8.22	0.03817	*AMER1/NOG*	2
2	GO:0048704	embryonic skeletal system morphogenesis	0.25	94/18888	0.01	50.23	6.96	0.04312	*NOG*	1
2	GO:0048706	embryonic skeletal system development	0.25	128/18888	0.01	36.89	5.93	0.04676	*NOG*	1
2	GO:0009952	anterior/posterior pattern specification	0.25	219/18888	0.00	21.56	4.45	0.06119	*NOG*	1
2	GO:0003002	regionalization	0.25	429/18888	0.00	11.01	3.05	0.09321	*NOG*	1
2	GO:0007389	pattern specification process	0.25	474/18888	0.00	9.96	2.88	0.09866	*NOG*	1

Table S13. Ranking of isolated DEGs that were filtered out in the network clustering process. The disease-causing (causal) gene is indicated in the first column, followed by the score calculated for each DEG based on its distance from the causal gene. Norm. Rank: normalized rank.

Causal Gene	Score	Norm. Rank	Rank	Gene Symbol	Dataset
ENSG00000198947	1.00	6.42e-05	1	*DMD*	DMD_myot
ENSG00000198947	0.74	0.03	497	*ACKR3*	DMD_myot
ENSG00000198947	0.63	0.06	865	*ARHGAP20*	DMD_myot
ENSG00000198947	0.44	0.07	1158	*TEX14*	DMD_myot
ENSG00000198947	0.50	0.08	1208	*SIM2*	DMD_myot
ENSG00000198947	0.57	0.09	1386	*SLITRK5*	DMD_myot
ENSG00000198947	0.48	0.10	1491	*PDE3A*	DMD_myot
ENSG00000198947	0.44	0.10	1503	*LDB2*	DMD_myot
ENSG00000198947	0.49	0.10	1547	*TMEM182*	DMD_myot
ENSG00000198947	0.39	0.11	1674	*DPYSL5*	DMD_myot
ENSG00000198947	0.88	0.01	164	*ABCB11*	DMD_pcard
ENSG00000198947	0.84	0.01	220	*ZNF469*	DMD_pcard
ENSG00000198947	0.66	0.02	379	*CDR2L*	DMD_pcard
ENSG00000198947	0.66	0.03	470	*CDH12*	DMD_pcard
ENSG00000198947	0.63	0.03	510	*TBX18*	DMD_pcard
ENSG00000198947	0.73	0.04	550	*SHFL*	DMD_pcard
ENSG00000198947	0.66	0.04	593	*ABCA12*	DMD_pcard
ENSG00000198947	0.67	0.05	795	*JCAD*	DMD_pcard
ENSG00000198947	0.54	0.06	929	*FER1L6*	DMD_pcard
ENSG00000198947	0.63	0.06	954	*ARHGAP45*	DMD_pcard
ENSG00000198947	0.83	0.02	263	*PTPRN*	DMD_cfib
ENSG00000198947	0.66	0.03	470	*CDH12*	DMD_cfib
ENSG00000198947	0.54	0.06	891	*NEK9*	DMD_cfib
ENSG00000198947	0.55	0.08	1297	*UNC5C*	DMD_cfib
ENSG00000198947	0.44	0.11	1641	*LAMB1*	DMD_cfib
ENSG00000198947	0.41	0.12	1843	*SIM1*	DMD_cfib
ENSG00000198947	0.55	0.12	1871	*SLC46A3*	DMD_cfib
ENSG00000198947	0.39	0.15	2321	*STC1*	DMD_cfib
ENSG00000198947	0.37	0.15	2322	*USP2*	DMD_cfib
ENSG00000198947	0.29	0.19	2990	*BMP4*	DMD_cfib
ENSG00000198947	0.99	6.42e-04	10	*EPSTI1*	DMD_myob
ENSG00000198947	0.97	6.04e-03	94	*SCN5A*	DMD_myob
ENSG00000198947	0.85	8.09e-03	126	*DDIT4L*	DMD_myob
ENSG00000198947	0.86	8.35e-03	130	*EVI2A*	DMD_myob
ENSG00000198947	0.92	0.02	237	*ADRA1B*	DMD_myob
ENSG00000198947	0.63	0.04	647	*IL16*	DMD_myob
ENSG00000198947	0.57	0.06	863	*ACSS3*	DMD_myob
ENSG00000198947	0.63	0.06	865	*ARHGEF26*	DMD_myob
ENSG00000198947	0.60	0.06	903	*KIF1A*	DMD_myob
ENSG00000198947	0.56	0.06	908	*MACROD2*	DMD_myob

Table S14. Ranking of isolated DEGs that were filtered out in the network clustering process. The disease-causing (causal) gene is indicated in the first column, followed by the score calculated for each DEG based on its distance from the causal gene. Norm. Rank: normalized rank.

Causal Gene	Score	Norm. Rank	Rank	Gene Symbol	Dataset
ENSG00000064419	0.99	5.14e-04	8	*ST6GAL1*	LGMD_myob
ENSG00000064419	0.89	0.01	173	*P2RX5*	LGMD_myob
ENSG00000064419	0.66	0.03	394	*LPIN3*	LGMD_myob
ENSG00000064419	0.67	0.03	394	*TRPV2*	LGMD_myob
ENSG00000064419	0.75	0.03	511	*FOLR1*	LGMD_myob
ENSG00000064419	0.66	0.04	560	*EDNRB*	LGMD_myob
ENSG00000064419	0.83	0.04	566	*LOC102724594*	LGMD_myob
ENSG00000064419	0.69	0.04	611	*HS3ST3A1*	LGMD_myob
ENSG00000064419	0.70	0.04	635	*BCAM*	LGMD_myob
ENSG00000064419	0.96	0.04	645	*SRPK3*	LGMD_myob
ENSG00000064419	0.82	0.02	234	*EPHA1*	LGMD_pbmc
ENSG00000064419	0.84	0.05	764	*CHERP*	LGMD_pbmc
ENSG00000064419	0.61	0.05	854	*LGMN*	LGMD_pbmc
ENSG00000064419	0.54	0.06	858	*CHST15*	LGMD_pbmc
ENSG00000064419	0.51	0.08	1273	*CA12*	LGMD_pbmc
ENSG00000064419	0.51	0.09	1419	*NUDT12*	LGMD_pbmc
ENSG00000064419	0.54	0.10	1630	*MPZL2*	LGMD_pbmc
ENSG00000064419	0.43	0.12	1810	*TMEM178B*	LGMD_pbmc
ENSG00000064419	0.59	0.12	1836	*ADTRP*	LGMD_pbmc
ENSG00000064419	0.46	0.12	1903	*FAM13A*	LGMD_pbmc
ENSG00000147894	0.93	8.03e-03	125	*NREP*	ALS_iN_C9ORF72
ENSG00000147894	0.87	8.09e-03	126	*SPATA6*	ALS_iN_C9ORF72
ENSG00000147894	0.91	0.01	157	*MAP1LC3C*	ALS_iN_C9ORF72
ENSG00000147894	0.83	0.02	335	*TGFBRAP1*	ALS_iN_C9ORF72
ENSG00000147894	0.64	0.03	445	*ST8SIA1*	ALS_iN_C9ORF72
ENSG00000147894	0.65	0.04	690	*ZC4H2*	ALS_iN_C9ORF72
ENSG00000147894	0.52	0.07	1044	*DENND3*	ALS_iN_C9ORF72
ENSG00000147894	0.70	0.07	1062	*TMEM150C*	ALS_iN_C9ORF72
ENSG00000147894	0.53	0.08	1219	*CCND2*	ALS_iN_C9ORF72
ENSG00000147894	0.47	0.08	1318	*SYTL1*	ALS_iN_C9ORF72
ENSG00000089280	0.93	2.76e-03	43	*ZFP36L2*	ALS_fib_FUS
ENSG00000089280	0.94	3.72e-03	58	*HS3ST3A1*	ALS_fib_FUS
ENSG00000089280	1.00	3.98e-03	62	*JMJD6*	ALS_fib_FUS
ENSG00000089280	0.96	0.02	235	*VRK1*	ALS_fib_FUS
ENSG00000089280	0.80	0.02	247	*ZBTB1*	ALS_fib_FUS
ENSG00000089280	0.85	0.02	260	*SERTAD3*	ALS_fib_FUS
ENSG00000089280	0.71	0.02	343	*ADAMTS6*	ALS_fib_FUS
ENSG00000089280	0.69	0.05	702	*MN1*	ALS_fib_FUS
ENSG00000089280	0.71	0.05	738	*FNDC10*	ALS_fib_FUS
ENSG00000089280	0.58	0.06	875	*RSRP1*	ALS_fib_FUS

Table S15. List of differential expressed genes (DEGs) clustered for all DMD datasets (DMD_myot, DMD_pCard, DMD_cfib and DMD_myob). It includes the cluster (CL) identifier, the number of DEGs per cluster (#D), the name of the dataset and the Gene Symbol of each DEG.

CL	#D	Dataset	Gene Symbol
0	8	DMD_myot	*ACVRL1, SLIT3, BMP4, DCC, SOSTDC1, BMP2, ROBO2, MGP*
1	6	DMD_myot	*LRP1B, CLU, PLXNA4, WSCD1, ELN, STAB2*
2	5	DMD_myot	*H3C3, H2BC3, POLE2, CGAS, CTSK*
3	3	DMD_myot	*LRRC4C, PKP2, DSC2*
4	3	DMD_myot	*WNT2B, WNT11, SFRP5*
5	3	DMD_myot	*MYH2, MYH8, EFS*
6	3	DMD_myot	*DOK6, FLT1, FGF13*
7	3	DMD_myot	*TRHDE, EMILIN2, PLAC9*
0	32	DMD_pcard	*RNASEL, CDKN2B, TAGLN2, GRM1, CALB2, CALN1, HPCAL4, SLC34A2, FILIP1L, LOX, CD93, LTBP2, FBN1, FBLN5, FBLN2, EFEMP1, THBD, PRELP, COTL1, PCDH10, SLPI, RYR1, ALDH1A2, SEZ6L2, FBXO2, CHST15, BOC, ELN, CYBRD1, GPM6A, NTM, ANGPT2*
1	29	DMD_pcard	*LDHA, EGFR, DCN, CDH1, MYCN, ENO1, ICAM5, DDIT4, NDRG1, RGS4, CD248, PRKCB, SMAD7, MAPK13, BHLHE40, MBP, ANXA1, MAGEL2, S100A11, ADAMTS2, OSMR, PTPRT, TRPC4, BHLHE41, SPON2, SPARCL1, CDCP1, SLC7A7, PLEKHH2*
2	26	DMD_pcard	*PLAT, HGF, COL1A1, FN1, LOXL3, THBS2, MMP2, COL1A2, SPARC, MRC2, TFPI, BCAT1, COL11A1, COL3A1, TNC, ITGA2, VWF, TNFRSF21, ITGA4, ITGA1, SMOC2, TGFBI, PLA2R1, HHIP, CNTN6, SERPINE2*
3	23	DMD_pcard	*RPL3L, S100A10, CRIM1, AHNAK2, AMIGO2, IL31RA, PTGS1, PTGIS, KCNA5, KCNB1, CBLN2, EMILIN1, COL26A1, COL9A3, COL6A3, COL6A6, MUC16, F2RL2, PROCR, CCDC80, SDR42E1, GCNT1, PDYN*
4	18	DMD_pcard	*PDGFRB, KDR, CD9, CAPN6, PODXL, PTPRQ, LUM, MMP14, SCARA3, ATP2B2, NHERF1, SERPINF1, IL1R1, DOK5, TENM2, FNDC1, DHRS9, OLFML1*
5	18	DMD_pcard	*RHOU, DSC3, SPTBN2, PDLIM3, EHBP1L1, MYH11, BDNF, DOCK11, SLC2A1, KCNMA1, ACTG2, NEB, TRIM38, MYH8, DAAM2, CGN, ITGA8, BST1*
6	18	DMD_pcard	*EPPK1, NPR3, MYLK, PRKG2, SLC35F2, ITGB4, GNAL, HLA-B, DRD1, LPAR1, VIPR1, GNG2, NNMT, CCL2, HMCN1, KCTD16, NPY, INMT*
7	16	DMD_pcard	*NELL2, NR2F1, COLEC11, ENG, NOTCH3, EGFL7, RXFP1, BMP4, GPC3, GLIPR1, POU2F2, BMP2, GDF2, TMEM108, GDF7, C1QTNF6*
8	15	DMD_pcard	*FOS, TBX3, FOXC1, TLE1, SP100, CDC42EP1, MAF, RUNX1, SEL1L3, PLTP, DACH1, NFE2L3, FLI1, ELK3, ELF4*
9	13	DMD_pcard	*LRP2, SHTN1, ADAM12, MEGF10, IGF2, KRT7, PRSS23, DKK2, DKK1, IGFBP3, PEAR1, IL33, IGF1*
10	10	DMD_pcard	*VCAN, HAPLN1, SLITRK2, PTPRF, DPP4, THY1, PTPRZ1, CXCL14, CXCL12, FAP*
11	9	DMD_pcard	*NUPR1, PFKFB3, KRT19, SNTB1, DMD, LIMA1, KRT80, GPRC5A, PFKFB4*
12	6	DMD_pcard	*APOB, MAP3K21, BGN, TLR4, TRAF5, CD36*
13	5	DMD_pcard	*C1RL, CFI, C3, SERPING1, CFH*
14	3	DMD_pcard	*SEMA3F, NRP2, PKHD1L1*
15	3	DMD_pcard	*LMO3, MN1, LHX9*
16	3	DMD_pcard	*SULF2, SULF1, MFAP5*
0	4	DMD_cfib	*ANLN, UACA, DMD, CD55*
1	3	DMD_cfib	*DCN, NTNG1, LUM*
2	3	DMD_cfib	*CDKN2B, CYTB, ANKRD6*
3	3	DMD_cfib	*VWA1, PCDHA10, PCDHA6*
0	32	DMD_myob	*SORCS2, TFRC, SPTB, KIF14, DPYSL5, MAP7, OAS3, BST2, CD9, MKI67, CEP55, CENPF, PKP2, GPAT3, ESCO2, APBA2, ASPM, ACTN3, ATP2A1, ASB4, BCHE, HELZ2, KNL1, SERPINB7, SP110, ANO2, NQO2, FHAD1, EFHD1, IFI44, EFR3B, CTHRC1*
1	29	DMD_myob	*ND4L, ND1, CYTB, COX2, RCCD1, IFI27, ND4, ROS1, MAP3K9, SHROOM2, SAMD9L, SCARA3, ITGB2, COX1, ND5, ND2, TTC24, SAMD9, SEZ6, NPIPB11, IFI6, NEU4, COL24A1, ND6, COX3, ADGRD1, SOD3, POSTN, TGFBI*
2	25	DMD_myob	*PLK1, FBN2, RRM2, PPP2R2B, IFIH1, KY, RIGI, PRKG2, LDOC1, RASGRF1, IFIT3, IFIT1, IFIT2, PNMA8A, ERC2, FBXL16, CCNO, RNF125, ARC, GUCY1A2, BCAS1, PALM, SKA1, RTL3, RIMS2*
3	23	DMD_myob	*IFITM3, TERT, COL22A1, H4C6, KIF11, SIK1, MX1, KIF18B, H1-5, HELLS, PODXL, H3C3, H3C2, H3C1, H3C8, H3C7, H1-3, H2BC17, POU5F1B, H2BC7, NAP1L3, GPX7, H2BC6*
4	21	DMD_myob	*PRDX2, ESR1, DTX3L, STAT1, XAF1, ASS1, PARP14, PBX1, LMO2, ARNT2, BIRC3, FOXM1, HOXA11, DEPDC1, HOXB9, IRX5, PARP9, HOXB13, LCNL1, MAB21L1, HOXA13*
5	21	DMD_myob	*PTPRD, BAALC, STK26, ADGRL3, LUM, LRRN1, FLRT2, ADGRA1, SLIT2, SLITRK5, PODNL1, PTPN13, FLT4, TEK, GLI4, DOK6, TENM3, GHR, FGF7, TENM4, ADGRF5*
6	20	DMD_myob	*LRRK2, AP1G2, GAREM1, CIB2, MYO5C, MAMSTR, STK32B, MYH1, ACTN2, ACTA2, GSTM2, IQGAP3, CHGB, ACTG2, EPS8L2, MYH2, ATP6, ATP8, CCDC144A, GSTM1*
7	20	DMD_myob	*LRP4, DCX, AGRN, PYGM, THBS2, MMP2, DMD, TTYH2, AGTR1, S100A1, PCSK9, SIRPA, PCOLCE, CKMT2, S1PR1, LIN7A, KCNJ12, CYTIP, FAP, C11orf87*
8	19	DMD_myob	*SLC15A3, FREM2, OASL, ISG15, NFATC4, MAPK15, KCNQ5, IFI35, SELENOP, HERC5, SBSPON, DDX60, NDP, FBXO16, DUSP26, DUSP15, BATF2, MCOLN3, ZDHHC15*
9	11	DMD_myob	*DYNC1I1, GRM4, CCDC8, TOP2A, AMOT, PLEKHG3, EMILIN1, CPNE8, FAM131B, SEPTIN1, MFAP2*
10	8	DMD_myob	*SHANK1, IL20RA, DIRAS2, DIRAS1, KALRN, DLGAP1, PLCH1, PLCL1*
11	7	DMD_myob	*PCDH10, PCDHGA12, PCDHGA10, PCDHGB2, PCDHGB4, PCDHB2, PCDHGB6*
12	3	DMD_myob	*VANGL2, SFRP1, FZD3*

Table S16. List of differential expressed genes (DEGs) clustered for all LGMD (LGMD_myob and LGMD_pbmc) and ALS (ALS_iN_C9ORF72, ALS_fib_FUS) datasets. It includes the cluster (CL) identifier, the number of DEGs per cluster (#D), the name of the dataset and the Gene Symbol of each DEG.

CL	#D	Dataset	Gene Symbol
0	29	LGMD_myob	*SEMA3G, SEMA6A, SEMA3A, SEMA5A, PLXNB3, SYT12, ATP1A2, TMC6, PCDHGB3, PKHD1, ATP2A3, BCL2, TMTC2, ATP2A1, FAIM2, CST6, PCDHB5, PCDHGA5, PCDHB3, PCDHGB4, VNN2, ATP1B4, SLN, EMILIN2, PCDHGB5, SEPTIN4, PLAC9, ST8SIA1, PCDHB4*
1	27	LGMD_myob	*DCN, C3orf52, CLEC11A, FBN2, GATD3, SMOC1, FBLN2, NTNG1, MXRA5, EPYC, LRRN1, FMOD, TPBG, LRRC15, CHRD, LAMA1, IGF2, F2R, F2RL2, TNC, PCOLCE, DDIT4L, FOXP2, ELN, CD36, ACAN, PROB1*
2	25	LGMD_myob	*ERBB3, SHD, MYL1, MYL11, MYL4, MYL3, MYL6B, PWP2, GRIA1, MYH6, EEF1A2, MYH1, MYH3, ACTC1, ACTN2, ACTG2, MYH2, MYH7, MYH8, FBXL22, UNC45B, MYOZ2, KCNN3, SGCG, SYNPO2L*
3	19	LGMD_myob	*ID1, FOS, HSD17B14, LDB3, MYOG, NFIA, KRT31, GYG2, MEF2C, SNAI1, NES, PIEZO2, PAX8, FOSB, MAFB, P2RX7, LHX4, ZDHHC15, CHRNG*
4	18	LGMD_myob	*KLHL40, AQP3, AQP1, TTN, OBSCN, CKB, PPM1E, PPM1L, XIRP2, NEB, LMOD3, TRIM54, PHOSPHO1, CKM, XIRP1, MYOM1, TSPAN33, ADAMTSL4*
5	16	LGMD_myob	*CORIN, VANGL2, AIF1L, TES, THRB, HRC, CASQ2, TRDN, RYR1, CSRP2, CAMK2B, JCHAIN, ANGPTL7, ANXA3, HMCN2, FZD9*
6	16	LGMD_myob	*COL22A1, MAPRE3, TUBB2B, SIK1, TAS1R1, DCLK1, VASH2, LOC102724428, AIRE, FBLN7, H3C8, H3C7, SERTAD4, POU4F1, SMYD1, L3MBTL1*
7	13	LGMD_myob	*CAPG, SCIN, TNNC1, TNNC2, ACTA1, TMOD1, TNNI1, TNNT1, TNNT3, TNNT2, NOX5, TNNI2, KISS1*
8	13	LGMD_myob	*FLT4, STYK1, CACNA1A, RAI2, SLCO4C1, CACNA1H, FGF13, DOK5, CACNA1S, CACNA1E, SCN4A, SCN7A, CACNG1*
9	7	LGMD_myob	*SHANK1, RERG, DIRAS2, SPTBN4, SPTB, EPB41, RAP1GAP2*
10	7	LGMD_myob	*LRP4, LDOC1, KREMEN2, APOL4, BCAS1, ST8SIA5, STAB2*
11	5	LGMD_myob	*ADRA1D, DPP4, FNDC5, ACKR3, CXCL12*
12	4	LGMD_myob	*PKP1, RNLS, FLG, ALDH3A1*
13	4	LGMD_myob	*COL21A1, COL4A6, COL4A5, C1QTNF1*
14	3	LGMD_myob	*RNF122, RSPO3, RSPO4*
0	6	LGMD_pbmc	*MMP9, CXCL2, CCL20, CXCL5, CXCL1, CXCL3*
1	5	LGMD_pbmc	*MYO5C, PALLD, CALD1, HLA-DQA1, HLA-DQB1*
2	4	LGMD_pbmc	*NLRP3, IL1R2, IL1B, IL1A*
3	4	LGMD_pbmc	*CXCL8, CCL2, CXCL9, CCL8*
0	35	ALS_iN_C9ORF72	*MAGI3, NELL2, TAGLN3, CALB1, RHBDL3, MYO5B, AK5, KIF5C, ENO2, FBLN1, EZR, NHS, NIBAN2, SYNE2, VCAN, EFEMP2, CRB2, ELAPOR2, DLG3, PODXL, LTBP1, PARM1, ACTC1, LCP1, SDC2, MRAP2, ABCA1, ACTL8, EFS, CCDC33, NEDD9, FILIP1, TIE1, BIK, MYCL*
1	27	ALS_iN_C9ORF72	*SOX2, KIF21B, IMPA2, EBF1, MEIS2, MYEF2, NAV2, MACROH2A2, MFGE8, EIF2S3B, SCN3B, HAL, PKP3, H2BC14, ASH2L, SERPINF1, SPINK5, ZNF462, ZNF608, SCD5, GLI3, FIGNL2, ENPP4, ST6GALNAC3, RBP1, SERPINB8, UCP2*
2	15	ALS_iN_C9ORF72	*SLC7A2, TTN, HLA-DRA, EEF1A2, MARCKS, MRC2, COL6A1, COL2A1, MFAP4, TMEM131L, COLGALT2, IGFN1, TSPAN5, CILP, CLEC1A*
3	13	ALS_iN_C9ORF72	*NANOG, PCDH15, BCAT1, TEX15, F2RL1, ARMCX4, ALPL, ARID3B, DCHS1, CDH7, CECR2, LRATD2, SALL4*
4	11	ALS_iN_C9ORF72	*SFN, MLLT11, RAB3IP, ARK2C, RAB15, P2RX5, CYP26C1, LEF1, DACT1, SMTNL2, ZNF154*
5	8	ALS_iN_C9ORF72	*CDH2, SRC, EPHB2, EPHA3, FMO5, MGAM, DDR2, TRIM7*
6	6	ALS_iN_C9ORF72	*MAGED2, ADGRG6, GUCY1B1, GNAL, FNDC5, NPR1*
7	6	ALS_iN_C9ORF72	*MPP2, TFPI, MEOX2, CNTNAP2, CNTN1, HHIP*
8	6	ALS_iN_C9ORF72	*RBPMS, RXRA, NCOA1, ZBTB16, SIM1, ZBTB32*
0	8	ALS_fib_FUS	*RIPK4, RHOBTB1, PANK1, TUBE1, KIF26B, RGS2, RGS4, IFIT2*
1	6	ALS_fib_FUS	*RND3, NOTCH1, DES, JUP, SLC12A7, CBARP*
2	4	ALS_fib_FUS	*IQCN, SLC25A25, AMER1, NOG*
3	3	ALS_fib_FUS	*HOXC6, HOXC4, HOXB5*
